# Development and validation of the FRAGIRE tool for assessment an older person’s risk for frailty

**DOI:** 10.1186/s12877-016-0360-9

**Published:** 2016-11-17

**Authors:** Dewi Vernerey, Amelie Anota, Pierre Vandel, Sophie Paget-Bailly, Michele Dion, Vanessa Bailly, Marie Bonin, Astrid Pozet, Audrey Foubert, Magdalena Benetkiewicz, Patrick Mankoundia, Franck Bonnetain

**Affiliations:** 1 Methodological and Quality of Life in Oncology Unit, INSERM U1098, University Hospital of Besançon, Besançon, France; 2 National clinical research Platform for Quality of life in Oncology, Besançon, France; 3 Department of psychiatry, EA 481, University Hospital of Besançon, Besançon, France; 4 Centre Georges Chevrier «Knowledge: norms and sensitivities», UMR CNRS 7366, University of Burgundy, Dijon, France; 5 Interregional Gerontology Pole from Burgundy and Franche-Comté, Dijon, France; 6 GERCOR, Groupe Coopérateur Multidisciplinaire en Oncologie, Paris, France; 7 Department of Geriatrics and Internal Medicine, Hospital of Champmaillot, University Hospital, Dijon, France; 8 Inserm/U1093 Cognition, Action and Sensorimotor Plasticity, University of Burgundy Franche-Comté, Dijon, France

**Keywords:** Elderly, Frailty, Loss of autonomy, Evaluation tool

## Abstract

**Background:**

Frailty is highly prevalent in elderly people. While significant progress has been made to understand its pathogenesis process, few validated questionnaire exist to assess the multidimensional concept of frailty and to detect people frail or at risk to become frail. The objectives of this study were to construct and validate a new frailty-screening instrument named Frailty Groupe Iso-Ressource Evaluation (FRAGIRE) that accurately predicts the risk for frailty in older adults.

**Methods:**

A prospective multicenter recruitment of the elderly patients was undertaken in France. The subjects were classified into financially-helped group (FH, with financial assistance) and non-financially helped group (NFH, without any financial assistance), considering FH subjects are more frail than the NFH group and thus representing an acceptable surrogate population for frailty. Psychometric properties of the FRAGIRE grid were assessed including discrimination between the FH and NFH groups. Items reduction was made according to statistical analyses and experts’ point of view. The association between items response and tests with “help requested status” was assessed in univariate and multivariate unconditional logistic regression analyses and a prognostic score to become frail was finally proposed for each subject.

**Results:**

Between May 2013 and July 2013, 385 subjects were included: 338 (88%) in the FH group and 47 (12%) in the NFH group. The initial FRAGIRE grid included 65 items. After conducting the item selection, the final grid of the FRAGIRE was reduced to 19 items. The final grid showed fair discrimination ability to predict frailty (area under the curve (AUC) = 0.85) and good calibration (Hosmer-Lemeshow *P*-value = 0.580), reflecting a good agreement between the prediction by the final model and actual observation. The Cronbach's alpha for the developed tool scored as high as 0.69 (95% Confidence Interval: 0.64 to 0.74). The final prognostic score was excellent, with an AUC of 0.756. Moreover, it facilitated significant separation of patients into individuals requesting for help from others (*P*-value < 0.0001), with sensitivity of 81%, specificity of 61%, positive predictive value of 93%, negative predictive value of 34%, and a global predictive value of 78%.

**Conclusions:**

The FRAGIRE seems to have considerable potential as a reliable and effective tool for identifying frail elderly individuals by a public health social worker without medical training.

**Electronic supplementary material:**

The online version of this article (doi:10.1186/s12877-016-0360-9) contains supplementary material, which is available to authorized users.

## Background

Frailty, a core geriatric concept, is considered highly prevalent and heterogeneous in its level of expression [1]. Most people aged 65 years or over lead independent live. However, as people age, progressively they are more likely to live with frailty. Twenty-five to 50% of elderly subjects older than 85 years old could be considered frail in the North American [1, 2] and European [3] countries. In the Survey of Health, Aging and Retirement in Europe (SHARE), the prevalence of frailty is estimated at 17% in Europe and 15% in France for people older than 65 years. Frailty represents therefore an important clinical and public health problem.

Significant progress has been made to understand its pathogenesis process and several definitions of this concept have been proposed. Despite a recent large interest on the subject, and various models, definitions, and criteria [4], frailty is still an evolving concept [5, 6]. Nevertheless, frailty has been acknowledged consensually as a multidimensional geriatric concept combining both health status and environmental components (including sociability, accommodation and transport accessibility), but also increased vulnerability and loss of adaptability to stress [4, 7]. Frailty has been demonstrated in various populations as a predictor of negative health outcomes, such as falls, hip fractures, worsening mobility, activities of daily living disability, need for long-term care, hospitalization, and mortality. Therefore, identification of older individuals who are frail or at risk of becoming frail with appropriate subsequent tailored evaluation and intervention constitutes an important goal of geriatric medicine [8]. Properly assessed frailty indicators could prevent the dependency and thereby could provide a better quality of life to this population and have large benefits for families and society [9]. Age-related functional decline is usually a slow process including a phase during which individuals at risk for frailty can be identified and referred for preventive interventions [10].

Currently, there are only few or not adequate tools to measure frailty or risk for frailty in the elderly people. In France, the Short Emergency Geriatric Assessment (SEGAm) seems to be the most interesting instrument, but it mainly detects frailty in elderly emergency conditions and it is not fully appropriate for geriatric assessment and in turns the risk of frailty [11]. Outside the emergency context, a widely used definition of frailty proposed by Fried et al. [1] considers frailty as similar to disability, comorbidity, and other characteristics and defines it as a clinical syndrome in which three or more of the following criteria are present: unintentional weight loss, self-reported exhaustion, reduction of grip strength, slow walking speed, and low physical activity. Fried’s phenotype model could provide important information but fails to provide a complete assessment and to predict the occurrence of frailty in the general elderly population who are not yet frail [6, 12]. The frailty index, defined by a cumulative deficit approach, has emerged as a promising concept in gerontology research [13]. Rockwood deficits accumulation model is based on the idea that the frailty is measured by the number of health problems associated with age, regardless of their nature and severity. This approach is a well-recognized tool and could be described as an overall indicator of health condition of the elderly people. Nevertheless, frailty index does not refer to a clearly defined conceptual model. It is also not an equivalent method of a comprehensive geriatric assessment as practiced in medico-social situations that is structured, standardized and focused on the identification of needs for assistance and care. A recent study provides a short review of the multidimensional frailty assessments that are currently available and concluded that Comprehensive Model of Frailty should ideally be a multidimensional and multidisciplinary construct including physical, cognitive, functional, psychosocial/family, environmental, and economic factors [14].

In this context, two French institutions for the elderly people, the National Old-Age Insurance Fund (The Caisse Nationale d'Assurance Vieillesse; CNAV) and the Central Fund of Social Agricultural Mutual (The Caisse Centrale de la Mutualité Sociale Agricole; CCMSA), have been stepping up efforts to assess a new multidimensional screening tool for frailty prediction in a specific population of older subjects autonomous in their daily life (Groupe Iso-Ressource (GIR) 5 and 6 [15, 16] that can be administered by social and other healthcare workers. The GIR 5 and 6 French populations are not a systematically helped population by public health funders, thus the identification of people at risk to become frail (i.e. to become a GIR 4 or lower elderly subject after some years) in this group of elderly could allow the prevention of the frailty with an adapted support of the institutions. A recently reported postal questionnaire in the INTER-FRAIL study [17] is one such tool, however this one focuses only on two domains: autonomy and activities of daily living (derived from the Katz’s index) [18]. The Fried’s frailty criteria, strongly centered on the physical and mobility dimensions, are also by definition not adapted for the GIR 5 and 6 population.

This article describes the development and validation of the Frailty GIR Evaluation (FRAGIRE), a new frailty-screening instrument to predict the risk of frailty in a specific GIR French elderly population not yet frail that can be administrated by a public health social worker without medical training. The FRAGIRE grid construction involves conventional factors (physical, cognitive, functional, psychosocial/family, and environmental) and other dimensions unexplored potentially interesting for contemporary frailty prediction in this population (cultural, sexual, and nutritional).

## Methods

### Participants

A prospective multicenter recruitment of older people (>60 years old) was undertaken between May 2013 and July 2013 in Bourgogne-Franche Comté, France. Patients belonged to the GIR 5 (people need occasional help with bathing, meal preparation and housekeeping) and 6 (people still autonomous for the main activities of daily life) groups of dependency (Additional file 1). Elderly subject in states GIR 5 and 6 cannot benefit from a systematic personal autonomy allowance from French institutions, but in particular situations they may receive a financial help of 3500 euros/year (pension additional plan [PAP]) for the following benefits: home care including cleaning, laundry, help with shopping and meal preparation; meal deliveries; little assistance with using the toilet, or home installation improvement. To be eligible for the PAP attribution elderly need to detail the motivation for such request. Whatever the amount of the retirement pension received, the elderly people could be eligible for the financial help weighted according to the pension received.

Patients selection was based on a hypothesis that the elderly in GIR 5 and 6 populations who claim the PAP, contrary to those who do not (the groups matched by age and gender), are probably more at risk to become frail and thus represent an acceptable surrogate population for frailty prediction.in GIR 5 and 6 population who are not yet frail. Based on this hypothesis, the subjects were classified into one of two groups: financially helped (FH, with financial assistance) group and non-financially helped (NFH, without any financial assistance) group.

The inclusion and exclusion criteria for each population are described in Additional file 2. Written consent was obtained from all subjects and the protocol was approved by the local ethics committee.

### Study design

**Fig. 1:**
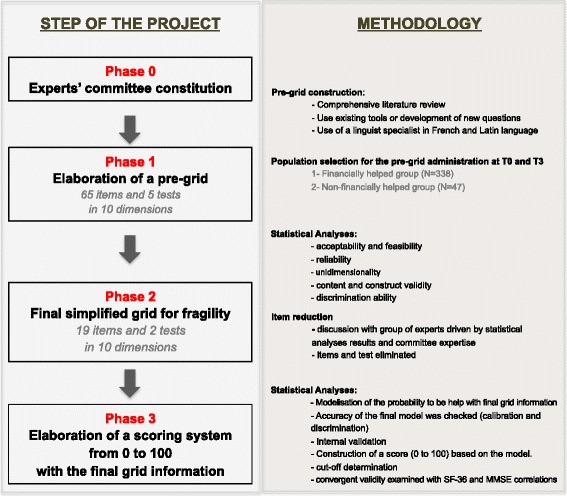
Study design: analysis and adaptation of the FRAGIRE model

The FRAGIRE grid was developed and validated in four phases with a cross-sectional cohort of elderly subjects (Fig. 1).

The first step, phases 0 and 1, was intended to provide the FRAGIRE pre-grid for an overall assessment of frailty including all potentially relevant items. This step was performed to ensure that all the frailty dimensions are captured and that data are collected for the second step. In the phase 0, a pluridisciplinary panel of expert committee was constituted. It consisted of a geriatrician, a psychiatrist, a demographer, a methodologist, an epidemiologist, a data manager, and the social support professionals. In the phase 1 (face validity), based on the experts’ knowledge about frailty and on a comprehensive literature review the FRAGIRE pre-grid with selected items was constructed. In order to cover a priori all-important fields of frailty and to warrant face and content validity of the pre-grid, number of items in the first step was not restricted.

The second analytic step, phases 2 and 3, aimed to assess the psychometrics properties of the FRAGIRE pre-grid, to reduce the number of items, to generate a frailty prognostic score to predict the probability of needing assistance from the French retirement aide system and thus by analogy the frailty based on the final FRAGIRE grid. In this step, criterion validity was also assessed by exploring the degree of concordance between the results from the final FRAGIRE grid and those of gold standards including the Medical Outcome Study Short Form-36 (SF-36) [19] and the Mini Mental State Examination (MMSE) [20]. The choice of items retained and construction of prognostic score was based on both psychometric properties analyses and experts’ recommendations. The following validation psychometrics parameters were assessed: construct validity of the general structure, dimensionality of the frailty variables with principal component analysis (PCA), convergent validity with the MMSE and SF-36 tools, discriminant validity (comparison of items response between the helped and the non-helped group), reliability including internal consistency (factorial analyses and Cronbach alpha coefficient calculations [21]), and repeatability/reproducibility (test-retest method).

### Data collection procedures and instruments

For each included subject, socio-demographic parameters were collected including age, gender, and job category in the pre-retirement period.

The FRAGIRE pre-grid was administered at inclusion (day 0). Items reproducibility was measured between two administrations of the pre-grid 3 days (maximum) apart. Majority of items were rated according to a 4-point Likert scale: 1) “not at all”, 2) “a little”, 3) “quite a bit”, and 4) “very much”.

In addition, participants were asked to fill out the SF-36 and MMSE questionnaires. The SF-36 is a 36-item well validated generic instrument measuring: physical functioning, role-physical, bodily pain, general health, vitality, social functioning, emotional role, and mental health. One score was generated per dimension on a 0–100 scale [19] with a high score reflects a high health-related quality of life level. The MMSE is a 30-item questionnaire evaluating various dimensions of cognition. The MMSE global score was generated as an index of global cognitive performance ranging from 0 to 30 (worst to best) [20]. Falls risks were assessed by the specific questionnaire, as per the recommendation of the French National Center of the Organization of Health Examination Centers (Centre Technique d'Appui et de Formation des Centre d'Examen de Santé [CETAF]). Questions were clearly enunciated to the elderly people and completed by a social worker according to the given responses (i.e. hetero-assessment). When an answer was not available in the item scale proposed, the social worker received the instruction to report a missing data.

In addition to the SF-36 and the MMSE, three other instruments were used. The Memory Impairment Screen (MIS) is a very brief 4-item screening tools for dementia. Patients score between 0 and 8 points, and a score of 5–8 is used to show no cognitive impairment while a score of less than 5 is used to show possible cognitive impairment [22]. The Isaacs Set Test (IST), consisting of generating a list of words (10 maximum) belonging to semantic categories in 15 s, evaluates verbal fluency abilities and speed of verbal production. Four semantic categories were successively used (cities, fruits, animals, and colors). A single score was generated ranges from 0 to 40, with higher score indicating better cognitive status [23]. The clock-drawing test (CDT) is a fast screening tool for cognitive impairment and dementia and can be used as a measure of spatial dysfunction and neglect [24].

Finally, the FRAGIRE pre-grid was reviewed with regard to clearness of the language, ambiguities, and ability of subject to understand the questionnaire without assistance.

### Sample size

The primary endpoint for questionnaire validation was reproducibility/repeatability using intraclass correlation coefficient (ICC) of the final score. Considering *a priori* introduced dimensions and *a* posteriori estimated ICC, the null hypothesis H0 of none agreement between two measurements was rejected if estimated ICC was 0.5 to and the alternative hypothesis H1 of reproducibility was accepted if the ICC of was at least 0.65. The type I error rate was fixed to 0.001 (Bonferroni correction, bilateral situation) and a statistical power to 80%. It was required to include at least 338 subjects. Test-retest reliability of the FRAGIRE global score was finally evaluated by ICC at an alpha type I error rate fixed at 0.05. For all other analyses, *P* < .05 was considered statistically significant.

### Statistical analysis

Mean (standard deviation) or median (range) values and frequencies (percentages) were provided for the description of continuous and categorical variables, respectively. The two groups were compared for means, medians, and proportions using Student’s t-test, non-parametric Mann–Whitney test, and chi-square test (or Fisher’s exact-test, if appropriate), respectively. The main psychometrics properties of the FRAGIRE pre-grid were evaluated using both classical tests and item response theory (IRT). Acceptability and feasibility were assessed regarding response rates and missing values. The construct validity and dimensional structure of the questionnaire were assessed using both PCA and IRT. Items of low clinical added value to dimension information were eliminated during the reduction phase, examining correlations between the item scores and dimension. A partial credit model by dimension derived from IRT model [25] will be reported elsewhere. Item-discriminant ability between the FH and the NFH group was assessed using Mann–Whitney test by comparing item response categories between groups. If a significant difference between items distribution among populations was observed, the item discrimination ability was supported. The PCA correlation circle also exhibited the items discrimination ability (contribution to the PC axes) and allowed us to visualize how they mutually interact (correlation). Reliability was evaluated by investigating both internal consistency and repeatability of the FRAGIRE measure using Cronbach’s alpha coefficients, which were computed across items to estimate the global internal consistency reliability and the internal consistency of each dimension. An alpha coefficient of 0.70 or higher was considered as acceptable [21, 26]. Uncertainties around Cronbach’s alpha coefficients were measured with a bootstrapping with calculation of a 95% confidence interval (95% CI). Repeatability was assessed by investigating changes in items response categories from day 0 to day 3 using Wilcoxon non-parametric test. An item was excluded if it demonstrated: missing value exceeding 10% (suggesting that subject had difficulty responding to the item); no discrimination ability, no added value in PCA, two items presenting quasi-complete positive or negative correlation (opposed on the PCA) induce the deletion of one item, and/or limited role in PCA correlation circle. Items were selected into the final grid based on the following criteria: high discrimination ability, large or acceptable contribution to PCA correlation circle, or clinically relevant items based on the choice of the expert group. The psychometrics properties of the final FRAGIRE grid were assessed after the item reduction phase.

For the phase 3, a global scoring system based on the selected items of the final FRAGIRE grid was developed, with items and tests as continuous variables. The association between items response and tests with “help requested status” was assessed in univariate and multivariate unconditional logistic regression analyses.

The predictive value and the discrimination ability [27] of the final model was evaluated with area under the curve (AUC) index, while calibration and goodness of fit of the model were assessed using Hosmer-Lemeshow test (i.e. the ability to provide unbiased predictions in groups of similar people). A high *P*-value (>0.1) was considered as an indicator for acceptable calibration. Bootstrapping [28] was used for internal validation of the model.

A score to predict help requested status was constructed and weighted with beta coefficients estimations from the final multivariate regression model. The possible changes in parameters were taken into account when the expert group suggests it. A prognostic score between 0 and 100 to predict the probability of needing assistance from the French retirement aide system and thus by analogy the frailty based on the final full model was calculated for each individual The FRAGIRE prognostic score, calculated for each subject, was normalized on a 0 to 100 scale with the highest score representing the most frail. A receiver operating characteristic (ROC) curve was constructed, with calculation of the AUC, to check discriminant capability of the score. The Youden index was used to identify the optimal threshold value [29]. Repeatability of prognostic score was also assessed by ICCs [30] Linear regression and Pearson’s coefficient correlation between the prognostic score at day 0 and day 3 were also computed. All analyses were performed using SAS version 9.3 (SAS Institute) and R software version 2.15.2 (R Development Core Team).

## Results

The characteristics of the two population groups (FH and NFH) are presented in Table 1. Overall, 385 retired elderly subjects, 338 (88%) in the FH group and 47 (12%) in the NFH group, were included.

**Table 1:** Baseline characteristics comparison between the two groups of patients (*N* = 385)

Characteristics	Total N (%)	Not-helped N (%)	Helped N (%)	*P*-value
Overall population	385	47 (12.2)	338 (87.8)	
Population after exclusion of patients with a GIR score of 4	383	47	336	
Age				
Mean ± SD (range)	81.9 ± 5.89 (63–94)	80.46 ± 4.87 (70–93)	82.11 ± 5.99 (63–94)	
Missing	2	0	2	
Sex				
Male	65 (16.88)	11 (23.4)	54 (16.0)	0.2
Female	320 (83.12)	36 (76.6)	284 (84.0)	
GIR score				
5	74 (19.2)	2 (4.3)	72 (21.3)	0.001
6	200 (52.0)	34 (72.3)	166 (49.1)	
Missing	111 (28.8)	11 (23.4)	100 (29.6)	
Marital status				
Single	25 (6.5)	2 (4.3)	23 (6.8)	0.001
Married	110 (28.6)	26 (55.3)	84 (24.8)	
Separated/Divorced	35 (9.1)	3 (6.4)	32 (9.5)	
Widow	212 (55.1)	16 (34.0)	196 (58.0)	
Missing	3 (0.7)	0	3 (0.9)	
Education				
Primary school	260 (67.5)	22 (46.8)	238 (70.4)	0.002
High school	49 (12.7)	10 (21.3)	39 (11.5)	
Vocational education	8 (2.1)	4 (8.5)	4 (1.2)	
High school plus 2 years of higher education	5 (1.3)	0	5 (1.5)	
Higher education	0	0	0	
Unknown education level	6 (1.6)	0	6 (1.8)	
Missing	57 (14.8)	11 (23.4)	46 (13.6)	
Socio-professional category				
Farmer	37 (9.6)	0	37 (11.0)	0.04
Artisans, merchants and business leader	23 (6.0)	2 (4.3)	21 (6.2)	
Managers and intellectual professions	7 (1.8)	1 (2.1)	6 (1.8)	
Middle-level occupations	24 (6.2)	4 (8.5)	20 (5.9)	
Employees	155 (40.3)	21 (44.7)	134 (39.6)	
Laborers	107 (27.8)	15 (31.9)	92 (27.2)	
Without occupational activity	18 (4.7)	4 (8.5)	14 (4.1)	
Unclassifiable	2 (0.5)	0	2 (0.6)	
Missing	12 (3.1)	0	12 (3.6)	
Department of residence				
Côte d'Or	45 (11.7)	8 (17.0)	37 (10.9)	0.002
Doubs	57 (14.8)	9 (19.1)	48 (14.2)	
Jura	67 (17.4)	6 (12.8)	61 (18.1)	
Nièvre	23 (6.0)	10 (21.3)	13 (3.8)	
Haute Saône	52 (13.5)	3 (6.3)	49 (14.5)	
Saône et Loire	95 (24.7)	7 (14.9)	88 (26.0)	
Yonne	32 (8.3)	2 (4.3)	30 (8.9)	
Territoire de Belfort	10 (2.6)	2 (4.3)	8 (2.4)	
Missing	4 (1.0)	0	4 (1.2)	
Region of residence				
Bourgogne	195 (50.6)	27 (57.4)	168 (49.7)	0.38
Franche Comté	186 (48.3)	20 (42.6)	166 (49.1)	
Missing	4 (1.0)	0	4 (1.2)	

*GIR* Iso-Resource Groups score

### The FRAGIRE pre-grid

For the phase 1, 65 items (Q1–Q65) describing 10 dimensions were identified (see Additional file 3): overall health status (4 items), emotional dimension (15 items), cognitive impairment (2 items plus 5 tests), environmental (9 items), cultural (2 items), sexual (4 items), burden of help (3 items), nutritional (8 items), neurosensory (6 items), mobility (9 items with 1 test), and proxy assessment of frailty by the social worker (3 items). This step resulted in a 65-item and 3-test grid (tests related to cognitive dimension: MIS, IST, and CDT) that administration lasted approximately 45 min. Tables 2 and 3 display the items of the FRAGIRE pre-grid and the distribution of responses rates. Most items have a large majority of responses. The maximal missing-item rates were 18% on day 0 and 21% on day 3. The items Q18, Q23, and Q39 were unanswered on day 0 by 16, 16, and 18% of subjects, respectively (Tables 2 and 3).

**Table 2:** The FRAGIRE pre-specified grid for dimension scores on day 0

Dimension	Measure	Questionnaire Item		Total		Non-financially helped group		Financially helped group		*P*-value
				*N* = 385		*N* = 47		*N* = 338		
				N	%	N	%	N	%	
General health status	Health status	Q1	Mean ± SD	5.7 ± 1.61		6.6 ± 1.8		5.5 ± 1.5		< .0001
			Missing	6		0		6		
	Health status compared to people of the same age group	Q2	Mean ± SD	5.7 ± 1.8		6.8 ± 2.0		5.5 ± 1.7		< .0001
			Missing	21		3		18		
	More than 5 medication per day	Q3	No	165	42.97	27	57.45	138	40.95	
			Yes	218	56.77	20	42.55	198	58.75	
			Don’t know	1	0.26	0		1	0.30	.088
			Missing	1		0		1		
	Number of hospitalizations within the last 6 months	Q4	0	275	72.37	36	76.60	239	71.77	
			1 - 2	93	24.47	9	19.15	84	25.23	
			More than 2	12	3.16	2	4.26	10	3.00	.619
			Missing	5				5		
Psychological	General well-being	Q5	Mean ± SD	5.9 ± 1.8		7.0 ± 1.7		5.7 ± 1.8		< .0001
			Missing	4		0		4		
	Spirit	Q6	Mean ± SD	5.9 ± 2.2		7.1 ± 1.8		5.8 ± 2.2		< .0001
			Missing	4		0		4		
	Unhappiness and depression	Q7	Not at all	160	41.67	23	48.94	137	40.65	
			A little	157	40.89	21	44.68	136	40.36	
			Quite a bit	49	12.76	2	4.26	47	13.95	
			Very much	18	4.69	1	2.13	17	5.04	.199
			Missing	1		0		1		
	Happiness	Q8	Not at all	24	6.32	2	4.26	22	6.61	
			A little	109	28.68	6	12.77	103	30.93	
			Quite a bit	210	55.26	33	70.21	177	53.15	
			Very much	37	9.74	6	12.77	31	9.31	.036
			Missing	5				5		
	Life satisfaction	Q9	Not very	160	41.99	24	51.06	136	40.72	
			Little	94	24.67	9	19.15	85	25.45	
			Pretty	102	26.77	13	27.66	89	26.65	
			Very much	25	6.56	1	2.13	24	7.19	.343
			Missing	4				4		
	Discouragement and sadness	Q10	Not at all	105	27.63	20	42.55	85	25.53	
			A little	185	48.68	20	42.55	165	49.55	
			Quite a bit	65	17.11	6	12.77	59	17.72	
			Very much	25	6.58	1	2.13	24	7.21	.074
			Missing	5				5		
	Positive consideration of life	Q11	Not at all	22	5.80	1	2.13	21	6.33	
			A little	132	34.83	12	25.53	120	36.14	
			Quite a bit	167	44.06	23	48.94	144	43.37	
			Very much	58	15.30	11	23.40	47	14.16	.160
			Missing	6				6		
	Usefulness	Q12	Not at all	36	9.40	2	4.26	34	10.12	
			A little	72	18.80	11	23.40	61	18.15	
			Quite a bit	158	41.25	19	40.43	139	41.37	
			Very much	117	30.55	15	31.91	102	30.36	.541
			Missing	2				2		
	Motivation to pursue leisure and usual activities	Q13	Not at all	62	16.23	4	8.51	58	17.31	
			A little	107	28.01	9	19.15	98	29.25	
			Quite a bit	144	37.70	21	44.68	123	36.72	
			Very much	69	18.06	13	27.66	56	16.72	.075
			Missing	3				3		
	Tension, anger, stress	Q14	Not at all	87	22.66	9	19.15	78	23.15	
			A little	127	33.07	18	38.30	109	32.34	
			Quite a bit	113	29.43	17	36.17	96	28.49	
			Very much	57	14.84	3	6.38	54	16.02	.246
			Missing	1				1		
	Difficulty sleeping	Q15	Not at all	128	33.51	23	48.94	105	31.34	
			A little	95	24.87	7	14.89	88	26.27	
			Quite a bit	88	23.04	10	21.28	78	23.28	
			Very much	71	18.59	7	14.89	64	19.10	.093
			Missing	3				3		
	Tiredness	Q16	Not at all	50	13.05	14	29.79	36	10.71	
			A little	152	39.69	16	34.04	136	40.48	
			Quite a bit	113	29.50	13	27.66	100	29.76	
			Very much	68	17.75	4	8.51	64	19.05	.002
			Missing	2				2		
	Enjoyment of daily activities	Q17	Not at all	30	7.83	4	8.51	26	7.74	
			A little	108	28.20	7	14.89	101	30.06	
			Quite a bit	177	46.21	23	48.94	154	45.83	
			Very much	68	17.75	13	27.66	55	16.37	.092
			Missing	2				2		
	Positive view of life	Q18	Not at all	19	5.86			19	6.74	
			A little	117	36.11	9	21.43	108	38.30	
			Quite a bit	132	40.74	18	42.86	114	40.43	
			Very much	56	17.28	15	35.71	41	14.54	.002
			Missing	61		5		56		
	Suicide ideation	Q19	Not at all	354	92.43	46	97.87	308	91.67	
			A little	26	6.79	1	2.13	25	7.44	
			Quite a bit	2	0.52	0		2	0.60	
			Very much	1	0.26	0		1	0.30	.479
			Missing	2		0		2		
Cognitive impairment	Difficulty concentrating	Q20	Not at all	229	59.48	35	74.47	194	57.40	
			A little	96	24.94	9	19.15	87	25.74	
			Quite a bit	47	12.21	1	2.13	46	13.61	
			Very much	13	3.38	2	4.26	11	3.25	.062
			Missing	0		0		0		
	Difficulty remembering	Q21	Not at all	96	25.00	15	31.91	81	24.04	
			A little	204	53.13	28	59.57	176	52.23	
			Quite a bit	60	15.63	2	4.26	58	17.21	
			Very much	24	6.25	2	4.26	22	6.53	.102
			Missing	1				1		
Environmental	Caregivers support	Q22	No	46	12.57	8	17.78	38	11.84	
			Yes	320	87.43	37	82.22	283	88.16	.334
			Don’t know							
			Missing	19		0		17		
	Satisfaction of support	Q23	Not at all	12	3.41	3	7.50	9	2.88	
			A little	21	5.97	3	7.50	18	5.77	
			Quite a bit	117	33.24	13	32.50	104	33.33	
			Very much	202	57.39	21	52.50	181	58.01	.372
			Missing	33		7		26		
	Feeling of loneliness/abandonment	Q24	Not at all	215	56.58	35	74.47	180	54.05	
			A little	120	31.58	10	21.28	110	33.03	
			Quite a bit	34	8.95	1	2.13	33	9.91	
			Very much	11	2.89	1	2.13	10	3.00	.049
			Missing	5				5		
	Contact with other impaired patients	Q25	Not at all	290	75.72	38	80.85	252	75.00	
			A little	60	15.67	5	10.64	55	16.37	
			Quite a bit	24	6.27	2	4.26	22	6.55	
			Very much	9	2.35	2	4.26	7	2.08	.478
			Missing	2				2		
	Missing activities	Q26	No	189	49.48	25	53.19	164	48.96	
			Yes	193	50.52	22	46.81	171	51.04	.642
			Don’t know							
			Missing	3				3		
	Envy of going out	Q27	No	98	25.72	12	25.53	86	25.75	
			Yes	278	72.97	35	74.47	243	72.75	
			Don’t know	5	1.31	0		5	1.50	1
			Missing	4				4		
	Satisfaction with mode of transportation	Q28	No	39	10.18	2	4.26	37	11.01	
			Yes	341	89.03	45	95.74	296	88.10	
			Don’t know	3	0.78	0		3	0.89	.347
			Missing	2				2		
	Financial problems	Q29	Not at all	219	57.48	34	72.34	185	55.39	
			A little	109	28.61	12	25.53	97	29.04	
			Quite a bit	30	7.87	0		30	8.98	
			Very much	23	6.04	1	2.13	22	6.59	.037
			Missing	4				4		
	Sufficient financial resources	Q30	Not at all	102	26.91	5	10.64	97	29.22	
			A little	134	35.36	13	27.66	121	36.45	
			Quite a bit	139	36.68	28	59.57	111	33.43	
			Very much	4	1.06	1	2.13	3	0.90	.001
			Missing	6				6		
Cultural	Use of internet	Q31	Not at all	350	91.62	39	82.98	311	92.84	
			A little	16	4.19	0		16	4.78	
			Quite a bit	6	1.57	2	4.26	4	1.19	
			Very much	10	2.62	6	12.77	4	1.19	.0002
			Missing	3				3		
	Participation in activities	Q32	Not at all	266	69.82	33	70.21	233	69.76	
			A little	57	14.96	5	10.64	52	15.57	
			Quite a bit	56	14.70	9	19.15	47	14.07	
			Very much	2	0.52	0		2	0.60	.634
			Missing	4				4		
Sexual	Troubled by signs of weakening	Q33	Not at all	58	15.18	14	29.79	44	13.13	
			A little	153	40.05	20	42.55	133	39.70	
			Quite a bit	115	30.10	6	12.77	109	32.54	
			Very much	56	14.66	7	14.89	49	14.63	.005
			Missing	3				3		
	Troubled by signs of aging	Q34	Not at all	128	33.60	19	40.43	109	32.63	
			A little	136	35.70	18	38.30	118	35.33	
			Quite a bit	88	23.10	8	17.02	80	23.95	
			Very much	29	7.61	2	4.26	27	8.08	.476
			Missing	4				4		
	Positive self-image	Q35	Not at all	39	10.40	5	10.64	34	10.37	
			A little	140	37.33	13	27.66	127	38.72	
			Quite a bit	165	44.00	24	51.06	141	42.99	
			Very much	31	8.27	5	10.64	26	7.93	.459
			Missing	10				10		
	Interest in sexual activity	Q36	Not at all	326	86.70	36	78.26	290	87.88	
			A little	39	10.37	9	19.57	30	9.09	
			Quite a bit	9	2.39	1	2.17	8	2.42	
			Very much	2	0.53	0		2	0.61	.149
			Missing	9				8		
Burden of help	Helping other relatives	Q37	Not at all	257	67.10	24	51.06	233	69.35	
			A little	49	12.79	11	23.40	38	11.31	
			Quite a bit	31	8.09	4	8.51	27	8.04	
			Very much	46	12.01	8	17.02	38	11.31	.048
			Missing	2				2		
	Responsible of other relatives	Q38	Not at all	131	53.47	11	35.48	120	56.07	
			A little	26	10.61	4	12.90	22	10.28	
			Quite a bit	38	15.51	10	32.26	28	13.08	
			Very much	50	20.41	6	19.35	44	20.56	.037
			Missing	140		16		124		
	Difficulty with self-care	Q39	Not at all	105	33.23	15	40.54	90	32.26	
			A little	36	11.39	9	24.32	27	9.68	
			Quite a bit	23	7.28	2	5.41	21	7.53	
			Very much	13	4.11	1	2.70	12	4.30	
			Don’t concern	139	43.99	10	27.03	129	46.24	.046
			Missing	69		10		59		
Nutritional	Problems with taste	Q40	Not at all	336	87.73	42	89.36	294	87.50	
			A little	28	7.31	3	6.38	25	7.44	
			Quite a bit	10	2.61	0		10	2.98	
			Very much	9	2.35	2	4.26	7	2.08	.534
			Missing	2				2		
	Lack of appetite	Q41	Not at all	253	66.23	33	71.74	220	65.48	
			A little	81	21.20	9	19.57	72	21.43	
			Quite a bit	31	8.12	3	6.52	28	8.33	
			Very much	17	4.45	1	2.17	16	4.76	.901
			Missing	3		1		2		
	Reduced food intake	Q42	Not at all	221	58.01	25	54.35	196	58.51	
			A little	110	28.87	14	30.43	96	28.66	
			Quite a bit	36	9.45	5	10.87	31	9.25	
			Very much	14	3.67	2	4.35	12	3.58	.862
			Missing	4		1		3		
	Weight loss	Q43	Not at all	258	67.36	38	80.85	220	65.48	
			A little	80	20.89	4	8.51	76	22.62	
			Quite a bit	24	6.27	4	8.51	20	5.95	
			Very much	21	5.48	1	2.13	20	5.95	.058
			Missing	2				2		
	Number of dental consultations	Q44	0	230	60.05	18	38.30	212	63.10	
			1	99	25.85	21	44.68	78	23.21	
			More than 1	53	13.84	8	17.02	45	13.39	
			Don’t know	1	0.26	0		1	0.30	.005
			Missing	2				2		
	Frequent dental pain	Q45	No	334	87.43	41	87.23	293	87.46	
			Yes	48	12.57	6	12.77	42	12.54	1
			Missing	3				3		
	Capable to eat on its own	Q46	No	11	2.88	2	4.26	9	2.69	
			Yes	371	97.12	45	95.74	326	97.31	.632
			Missing	3				3		
	Denture	Q47	No	105	27.49	17	36.17	88	26.27	
			Yes	277	72.51	30	63.83	247	73.73	.165
			Missing	3				3		
Neurosensory	Deterioration in vision	Q48	Not at all	133	34.73	21	44.68	112	33.33	
			A little	126	32.90	12	25.53	114	33.93	
			Quite a bit	68	17.75	10	21.28	58	17.26	
			Very much	56	14.62	4	8.51	52	15.48	.245
			Missing	2				2		
	Need of glasses	Q49	No	310	81.15	36	76.60	274	81.79	
			Yes	72	18.85	11	23.40	61	18.21	.426
			Don't known							
			Missing	3				3		
	Hearing discomfort	Q50	Not at all	183	47.78	24	51.06	159	47.32	
			A little	110	28.72	13	27.66	97	28.87	
			Quite a bit	54	14.10	6	12.77	48	14.29	
			Very much	36	9.40	4	8.51	32	9.52	.984
			Missing	2				2		
	Hearing aid	Q51	No	332	87.14	38	82.61	294	87.76	
			Yes	49	12.86	8	17.39	41	12.24	.347
			Missing	4		1		3		
	Suitable hearing aid	Q52	No	15	25.42	3	30.00	12	24.49	
			Yes	38	64.41	7	70.00	31	63.27	
			Don’t know	6	10.17	0		6	12.24	.753
			Missing	326		37		289		
	Hearing impairment	Q53	No	218	62.11	29	65.91	189	61.56	
			Yes	128	36.47	15	34.09	113	36.81	
			Don’t know	5	1.42	0		5	1.63	.867
			Missing	34		3		31		
Mobility	Falls	Q54	0	261	68.32	38	80.85	223	66.57	
			1	69	18.06	4	8.51	65	19.40	
			More than 1	52	13.61	5	10.64	47	14.03	.117
			Don’t know			0		0		
			Missing	3				3		
	Physical difficulties	Q55	Not at all	43	11.32	10	21.28	33	9.91	
			A little	71	18.68	11	23.40	60	18.02	
			Quite a bit	110	28.95	15	31.91	95	28.53	
			Very much	156	41.05	11	23.40	145	43.54	.023
			Missing	5				5		
	Walking speed	Q56	> =1m/s	126	34.24	22	47.83	104	32.30	
			Between 0.65 and < 1m/s	112	30.43	16	34.78	96	29.81	
			<0.65m/s	130	35.33	8	17.39	122	37.89	.019
			Missing	17		1		16		
	Going to toilet on its own	Q57	No	36	9.45			36	10.75	
			Yes	345	90.55	46		299	89.25	.013
			Missing	4		1		3		
	Need help going to toilet	Q58	No	74	66.67	15	93.75	59	62.11	
			Yes	37	33.33	1	6.25	36	37.89	.019
			Missing	274		31		243		
	Difficulties shopping on its own	Q59	No	125	32.98	28	60.87	97	29.13	
			Yes	254	67.02	18	39.13	236	70.87	< .0001
			Don’t know							
			Missing	6		1		5		
	Need help shopping	Q60	No	46	16.25	13	44.83	33	12.99	
			Yes	237	83.75	16	55.17	221	87.01	< .0001
			Missing	102		18		84		
	Doing cleaning on its own	Q61	No	311	81.84	16	34.78	295	88.32	
			Yes	69	18.16	30	65.22	39	11.68	< .0001
			Don't know							
			Missing	5		1		4		
	Need help cleaning	Q62	No	23	6.73	11	40.74	12	3.81	
			Yes	319	93.27	16	59.26	303	96.19	< .0001
			Missing	43		20		23		
Section for examiner	Global health status	Q63	Mean ± SD	6.3 ± 1.7		7.2 ± 1.9		6.1 ± 1.6		< .0001
			Missing	4		0		4		
	Health status compared to people of the same age group	Q64	Mean ± SD	6.3 ± 1.8		7.3 ± 1.9		6.1 ± 1.7		< .0001
			Missing	4		0		4		
	Risk of deterioration	Q65	Mean ± SD	5.8 ± 1.9		6.4 ± 1.9		5.8 ± 1.9		.025
			Missing	5		0		5

**Table 3:** FRAGIRE pre-grid items distributions between the two measurements (on day 0 and day 3)

Dimension	Measure	Item	Interpretation	Overall population *N* = 385		Overall population N =385		*P* value
				Day 0		Day 3		
				N	%	N	%	
Global health status	Health status	Q1	Mean ± SD	5.7 ± 1.61		5.6 ± 1.6		.394
			Missing	6		14		
	Health status compared with people of the same age group	Q2	Mean ± SD	5.7 ± 1.8		5.7 ± 1.5		1
			Missing	21		25		
	More than 5 medications per day	Q3	No	165	42.97	163	43.94	
			Yes	218	56.77	208	56.06	
			Don’t know	1	0.26	0	0	.911
			Missing	1		14		
	Number of hospitalization within the last 6 months	Q4	0	275	72.37	272	73.51	
			1–2 times	93	24.47	87	23.51	
			More than 2	12	3.16	11	2.97	.944
			Missing	5		15		
Psychological	General well-being	Q5	Mean ± SD	5.9 ± 1.8		5.8 ± 1.7		.436
			Missing	4		19		
	Spirit	Q6	Mean ± SD	5.9 ± 2.2		6.0 ± 1.9		.506
			Missing	4		17		
	Unhappiness and depression	Q7	Not at all	160	41.67	149	40.16	
			A little	157	40.89	173	46.63	
			Quite a bit	49	12.76	30	8.09	
			Very much	18	4.69	19	5.12	.135
			Missing	1		14		
	Happiness	Q8	Not at all	24	6.32	18	4.86	
			A little	109	28.68	123	33.24	
			Quite a bit	210	55.26	201	54.32	
			Very much	37	9.74	28	7.57	.391
			Missing	5		15		
	Life satisfaction	Q9	Not at all	160	41.99	163	44.29	
			A little	94	24.67	115	31.25	
			Quite a bit	102	26.77	80	21.74	
			Very much	25	6.56	10	2.72	.011
			Missing	4		17		
	Discouragement and sadness	Q10	Not at all	105	27.63	113	30.62	
			A little	185	48.68	185	50.14	
			Quite a bit	65	17.11	50	13.55	
			Very much	25	6.58	21	5.69	.487
			Missing	5		16		
	Positive consideration of life	Q11	Not at all	22	5.80	18	4.90	
			A little	132	34.83	131	35.69	
			Quite a bit	167	44.06	181	49.32	
			Very much	58	15.30	37	10.08	.142
			Missing	6		18		
	Usefulness	Q12	Not at all	36	9.40	35	9.46	
			A little	72	18.80	64	17.30	
			Quite a bit	158	41.25	176	47.57	
			Very much	117	30.55	95	25.68	.319
			Missing	2		15		
	Motivation to pursue leisure and usual activities	Q13	Not at all	62	16.23	58	15.80	
			A little	107	28.01	118	32.15	
			Quite a bit	144	37.70	150	40.87	
			Very much	69	18.06	41	11.17	.0540
			Missing	3		18		
	Tension, anger stress	Q14	Not at all	87	22.66	71	19.09	
			A little	127	33.07	160	43.01	
			Quite a bit	113	29.43	106	28.49	
			Very much	57	14.84	35	9.41	.0134
			Missing	1		13		
	Difficulty sleeping	Q15	Not at all	128	33.51	134	36.02	
			A little	95	24.87	106	28.49	
			Quite a bit	88	23.04	72	19.35	
			Very much	71	18.59	60	16.13	.374
			Missing	3		13		
	Tireness	Q16	Not at all	50	13.05	43	11.59	
			A little	152	39.69	165	44.47	
			Quite a bit	113	29.50	117	31.54	
			Very much	68	17.75	46	12.40	.159
			Missing	2		14		
	Enjoyement of daily activities	Q17	Not at all	30	7.83	27	7.30	
			A little	108	28.20	109	29.46	
			Quite a bit	177	46.21	194	52.43	
			Very much	68	17.75	40	10.81	.046
			Missing	2		15		
	Positive view of life	Q18	Not at all	19	5.86	17	5.31	
			A little	117	36.11	116	36.25	
			Quite a bit	132	40.74	151	47.19	
			Very much	56	17.28	36	11.25	.126
			Missing	61		65		
	Suicide ideation	Q19	Not at all	354	92.43	351	94.86	
			A little	26	6.79	15	4.05	
			Quite a bit	2	0.52	3	0.81	
			Very much	1	0.26	1	0.27	.359
			Missing	2		15		
Cognitive impairment	Difficulty concentrating	Q20	Not at all	229	59.48	200	54.05	
			A little	96	24.94	122	32.97	
			Quite a bit	47	12.21	40	10.81	
			Very much	13	3.38	8	2.16	.088
			Missing	0		15		
	Difficulty remembering	Q21	Not at all	96	25.00	73	19.84	
			A little	204	53.13	232	63.04	
			Quite a bit	60	15.63	46	12.50	
			Very much	24	6.25	17	4.62	.054
			Missing	1		17		
Environmental	Caregivers support	Q22	No	46	12.57	36	10.32	
			Yes	320	87.43	312	89.40	
			Don’t know	0	0	1	0.29	.350
			Missing	19		36		
	Satisfaction of support	Q23	Not at all	12	3.41	12	3.45	
			A little	21	5.97	16	4.60	
			Quite a bit	117	33.24	116	33.33	
			Very much	202	57.39	204	58.62	.888
			Missing	33		37		
	Feeling if loneliness/abandonment	Q24	Not at all	215	56.58	218	59.08	
			A little	120	31.58	110	29.81	
			Quite a bit	34	8.95	29	7.86	
			Very much	11	2.89	12	3.25	.862
			Missing	5		16		
	Contact with other impaired patients	Q25	Not at all	290	75.72	274	74.05	
			A little	60	15.67	73	19.73	
			Quite a bit	24	6.27	15	4.05	
			Very much	9	2.35	8	2.16	.302
			Missing	2		15		
	Missing activities	Q26	No	189	49.48	176	47.96	
			Yes	193	50.52	188	51.23	
			Don’t know	0	0	3	0.82	.257
			Missing	3		18		
	Envy of going out	Q27	No	98	25.72	86	23.43	
			Yes	278	72.97	278	75.75	
			Don’t know	5	1.31	3	0.82	.589
			Missing	4		18		
	Satisfaction with mode of transportation	Q28	No	39	10.18	32	8.67	
			Yes	341	89.03	334	90.51	
			Don’t know	3	0.78	3	0.81	.854
			Missing	2		16		
	Financial problems	Q29	Not at all	219	57.48	200	54.20	
			A little	109	28.61	120	32.52	
			Quite a bit	30	7.87	28	7.59	
			Very much	23	6.04	21	5.69	.715
			Missing	4		16		
	Sufficient financial resources	Q30	Not at all	102	26.91	95	25.96	
			A little	134	35.36	146	39.89	
			Quite a bit	139	36.68	122	33.33	
			Very much	4	1.06	3	0.82	.607
			Missing	6		19		
Cultural	Use of internet	Q31	Not at all	350	91.62	338	91.60	
			A little	16	4.19	14	3.79	
			Quite a bit	6	1.57	4	1.08	
			Very much	10	2.62	13	3.52	.821
			Missing	3		16		
	Participation in activities	Q32	Not at all	266	69.82	248	67.39	
			A little	57	14.96	64	17.39	
			Quite a bit	56	14.70	54	14.67	
			Very much	2	0.52	2	0.54	.855
			Missing	4		17		
Sexual	Troubled by signs of weakening	Q33	Not at all	58	15.18	44	11.92	
			A little	153	40.05	160	43.36	
			Quite a bit	115	30.10	118	31.98	
			Very much	56	14.66	47	12.74	.446
			Missing	3		16		
	Troubled by signs of aging	Q34	Not at all	128	33.60	110	29.81	
			A little	136	35.70	159	43.09	
			Quite a bit	88	23.10	81	21.95	
			Very much	29	7.61	19	5.15	.15
			Missing	4		16		
	Positive self-image	Q35	Not at all	39	10.40	35	9.56	
			A little	140	37.33	143	39.07	
			Quite a bit	165	44.00	166	45.36	
			Very much	31	8.27	22	6.01	.648
			Missing	10		19		
	Interest in sexual activity	Q36	Not at all	326	86.70	311	85.21	
			A little	39	10.37	43	11.78	
			Quite a bit	9	2.39	10	2.74	
			Very much	2	0.53	1	0.27	.856
			Missing	9		20		
Burden of help	Helping other relatives	Q37	Not at all	257	67.10	254	68.65	
			A little	49	12.79	54	14.59	
			Quite a bit	31	8.09	22	5.95	
			Very much	46	12.01	40	10.81	.583
			Missing	2		15		
	Responsible of other relatives	Q38	Not at all	131	53.47	119	52.89	
			A little	26	10.61	19	8.44	
			Quite a bit	38	15.51	36	16.00	
			Very much	50	20.41	51	22.67	.8230
			Missing	140		160		
	Difficulties with self-care	Q39	Not at all	105	33.23	92	30.07	
			A little	36	11.39	38	12.42	
			Quite a bit	23	7.28	26	8.50	
			Very much	13	4.11	11	3.59	
			Don’t concern	139	43.99	139	45.42	.894
			Missing	69		79		
Nutritional	Problems with taste	Q40	Not at all	336	87.73	326	88.35	
			A little	28	7.31	27	7.32	
			Quite a bit	10	2.61	9	2.44	
			Very much	9	2.35	7	1.90	.977
			Missing	2		16		
	Lack of appetite	Q41	Not at all	253	66.23	240	65.22	
			A little	81	21.20	85	23.10	
			Quite a bit	31	8.12	30	8.15	
			Very much	17	4.45	13	3.53	.871
			Missing	3		17		
	Reduced food intake	Q42	Not at all	221	58.01	210	56.91	
			A little	110	28.87	121	32.79	
			Quite a bit	36	9.45	25	6.78	
			Very much	14	3.67	13	3.52	.453
			Missing	4		16		
	Weight loss	Q43	Not at all	258	67.36	253	68.38	
			A little	80	20.89	81	21.89	
			Quite a bit	24	6.27	21	5.68	
			Very much	21	5.48	15	4.05	.797
			Missing	2		15		
	Number of dental consultations	Q44	0	230	60.05	234	63.24	
			1	99	25.85	89	24.05	
			More than 1	53	13.84	46	12.43	
			Don’t know	1	0.26	1	0.27	.832
			Missing	2		15		
	Frequent dental pain	Q45	No	334	87.43	337	91.08	
			Yes	48	12.57	33	8.92	.126
			Missing	3		15		
	Capable to eat on its own	Q46	No	11	2.88	8	2.17	
			Yes	371	97.12	360	97.83	.644
			Missing	3		17		
	Denture	Q47	No	105	27.49	99	26.76	
			Yes	277	72.51	271	73.24	.870
			Missing	3		15		
Neurosensory	Deterioration in vision	Q48	Not at all	133	34.73	124	33.60	
			A little	126	32.90	138	37.40	
			Quite a bit	68	17.75	64	17.34	
			Very much	56	14.62	43	11.65	.489
			Missing	2		16		
	Need of glasses	Q49	No	310	81.15	310	84.01	
			Yes	72	18.85	58	15.72	
			Don’t know	0	0	1	0.27	.289
			Missing	3		16		
	Hearing discomfort	Q50	Not at all	183	47.78	181	48.92	
			A little	110	28.72	108	29.19	
			Quite a bit	54	14.10	56	15.14	
			Very much	36	9.40	25	6.76	.613
			Missing	2		15		
	Hearing aid	Q51	No	332	87.14	321	87.23	
			Yes	49	12.86	47	12.77	1
			Missing	4		17		
	Suitable hearing aid	Q52	No	15	25.42	15	24.59	
			Yes	38	64.41	41	67.21	
			Don’t know	6	10.17	5	8.20	.959
			Missing	326		324		
	Hearing impairment	Q53	No	218	62.11	212	63.28	
			Yes	128	36.47	121	36.12	
			Don’t know	5	1.42	2	0.60	.616
			Missing	34		50		
Mobility	Falls	Q54	0	261	68.32	249	68.03	
			1	69	18.06	67	18.31	
			More than 1	52	13.61	49	13.39	
			Don’t know	0	0	1	0.27	.977
			Missing	3		19		
	Physical difficulties	Q55	Not at all	43	11.32	35	9.54	
			A little	71	18.68	59	16.08	
			Quite a bit	110	28.95	124	33.79	
			Very much	156	41.05	149	40.60	.442
			Missing	5		18		
	Walking speed	Q56	> = 1 m/s	126	34.24	121	34.97	
			0.65 < 1 m/s	112	30.43	111	32.08	
			<0.65 m/s	130	35.33	114	32.95	.790
			Missing	17		39		
	Using toilet on its own	Q57	No	36	9.45	37	10.11	
			Yes	345	90.55	329	89.89	.806
			Missing	4		19		
	Need help going toilet	Q58	No	74	66.67	62	58.49	
			Yes	37	33.33	44	41.51	.261
			Missing	274		279		
	Difficulties shopping on its own	Q59	No	125	32.98	107	29.32	
			Yes	254	67.02	257	70.41	
			Don’t know	0	0	1	0.27	.304
			Missing	6		20		
	Need help shopping	Q60	No	46	16.25	50	17.73	
			Yes	237	83.75	232	82.27	.656
			Missing	102		103		
	Doing cleaning on its own	Q61	No	311	81.84	311	84.74	
			Yes	69	18.16	54	14.71	
			Don’t know	0	0	2	0.54	.147
			Missing	5		18		
	Need help cleaning	Q62	No	23	6.73	25	7.51	
			Yes	319	93.27	308	92.49	.765
			Missing	43		52		
Section for examiner	Global health status	Q63	Mean ± SD	6.3 ± 1.7		6.5 ± 3.9		.360
				4		17		
	Health status compared to people of the same age group	Q64	Mean ± SD	6.3 ± 1.8		6.3 ± 1.7		1
			Missing	4		19		
	Risk of deterioration	Q65	Mean ± SD	5.8 ± 1.9		5.9 ± 1.8		.461
			Missing	5		18		

The comparison of scores in each item, considering the FH and NFH groups, showed discrimination power (Mann–Whitney test *P* < 0.05) between the two groups with respect to 26 items (Q1, Q2, Q5, Q6, Q8, Q16, Q18, Q24, Q29, Q30, Q31, Q33, Q37, Q38, Q44, and Q55 to Q65).

Overall health, emotional, and separate examiner dimensions showed good internal consistency, with Cronbach’s alpha coefficient of > 0.80 (Table 4). Low internal consistency, with a coefficient of < 0.50, was associated with cognitive, cultural, sexual, and neurosensory dimensions. Internal consistency was high for the whole scale (a coefficient of 0.87). Wilcoxon non-parametric test of the FRAGIRE pre-grid showed good reproducibility between the two evaluations (on day 0 and on day 3) except for three items: Q9 (*P* = .011), Q14 (*P* = .013), and Q17 (*P* = .045).

**Table 4:** Cronbach alpha coefficient estimation before and after the-items-selection procedure

Dimension	Items	Before selection (Pre-grid)		After selection (Final grid)	
		Cronbach's alpha	95% Bootstrap CI	Cronbach's alpha	95% Bootstrap CI
General health status	Q1^†^, Q2	0.80	0.73–0.85	NA	NA
Psychological well-being	Q5^†^, Q6, Q7^a†^, Q8^†^, Q9 ^a^, Q10 ^a^, Q11, Q12, Q13^†^, Q14 ^a^, Q15 ^a^, Q16 ^a †^, Q17, Q19 ^a †^	0.82	0.79–0.84	0.66	0.60–0.71
Cognitive impairment	Q20 ^a †^, Q21 ^a †^	0.47	0.29–0.60	0.47	0.29–0.60
Environmental	Q24 ^a †^, Q25 ^a^, Q29 ^a^, Q30^†^	0.52	0.40–0.62	0.13	−0.08–0.31
Cultural	Q31^†^, Q32^†^	0.36	0.11–0.55	0.36	0.11–0.55
Sexual	Q33 ^a^, Q34 ^a †^, Q35, Q36^†^	0.38	0.24–0.49	0.03	−0.20–0.18
Burden of help		NA	NA	NA	NA
Nutritional	Q40 ^a †^, Q41 ^a †^, Q42 ^a^, Q43 ^a^	0.62	0.51–0.71	0.32	0.12–0.51
Neurosensory	Q48 ^a †^, Q50 ^a †^	0.33	0.11–0.48	0.33	0.11–0.48
Mobility	Q55 ^a †^, Q56	0.53	0.42–0.62	NA	NA
Section for examiner	Q63^†^, Q64, Q65^†^	0.86	0.82–0.89	0.73	0.65–0.80
Overall	Q1^†^, Q2, Q5^†^, Q6, Q7 ^a †^, Q8^†^, Q9 ^a^, Q10 ^a^, Q11, Q12, Q13^†^, Q14 ^a^, Q15 ^a^, Q16 ^a †^, Q17, Q19 ^a †^, Q20 ^a †^, Q21 ^a †^, Q24 ^a †^, Q25 ^a^, Q29 ^a^, Q30^†^, Q31^†^, Q32^†^, Q33 ^a^, Q34 ^a †^, Q35, Q36^†^, Q40 ^a †^, Q41 ^a †^, Q42 ^a^, Q43 ^a^, Q48 ^a †^, Q50 ^a^, Q55 ^a †^, Q56 ^a^, Q63^†^, Q64, Q65	0.86	0.84–0.88	0.69	0.64–0.73

^a^ is an item with reverse quotation; ^†^, Item selected in the final grid ; *CI* confidential interval, *NA* not available

Given the scoring heterogeneity (items scored as either 2 or 8 according to examiner) of the CDT and its poor observed compliance (53% and 58% of data available on day 0 and day 3, respectively), this test was no longer considered in the study.

A first stage of items selection process was based on completion rates and the extend of missing data on day 0 (Table 2). Eight items (12%; Q18, Q23, Q39, Q52, Q53, Q58, Q60, and Q62) were excluded at this stage. Five of those (Q52, Q53, Q58, Q60, and Q62) demonstrated a high rate of missing data due to the inter-item correlation therefore too difficult to handle in a scoring system. At a second stage of an elimination process (based on the item distribution comparison between the two groups (Table 2) and the PCA analysis of all dimensions made of at least two items [data not shown]), a total of 37 items were deleted due to: lack of discrimination ability (Q20, Q21, Q22, Q48, and Q50), lack of discrimination ability and no particular interest to PCA (Q26, Q27, Q28, and Q47), and lack of discrimination ability and presence of quasi-complete positive or negative correlation (Q7, Q9, Q11, Q12, Q13, Q14, Q15, Q17, Q25, Q35, Q41, Q42, Q45, Q46, Q49, and Q51). Moreover, eight items (Q2, Q6, Q10, Q29, Q33, Q43, Q59, and Q64) with almost complete correlation or rated as not relevant by a panel of experts were excluded despite their discrimination power. The final four items (Q3, Q57, Q61, and Q65) were removed due to their limiting role in PCA correlation circle. Two items, Q37 and Q38, composing “burden in help” dimension were combined in one single item in order to synthetize and simplify information from both items. The final set of items excluded were discussed and validated by a panel of experts.

### The final FRAGIRE grid

**Fig. 2:**
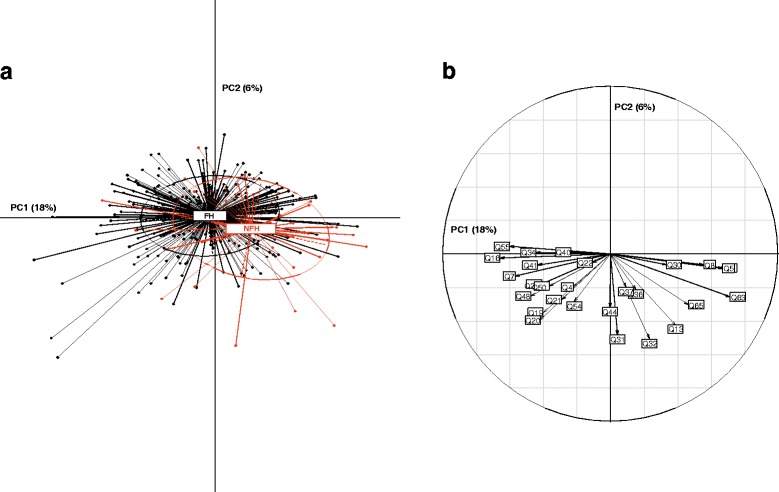
Principal component analysis with the items retained in the final FRAGIRE grid: Panel **a** shows the projection of individuals on the two principal component and Panel **b** shows the correlation circle providing the item’s interaction and contribution to the axes for component 1 and 2 on which the individual projection is made in the Panel **a**. Each axis can be considered as a linear combination of items weighted by their importance. Abbreviations: PC: Principal component; FH: financially helped group; NFH: Non-financially helped group

The selection process resulted in the final FRAGIRE grid composed of 19 items describing 9 dimensions (with examiner section) and 2 tests (see Additional file 4). Of 19 items, 11 (58%) had high discrimination ability and contribution in PCA correlation circle (Q1, Q5, Q8, Q16, Q24, Q30, Q31, Q44, Q55, Q56, and Q63), four (Q4, Q34, Q40, and Q54) had only an acceptable contribution in PCA correlation circle, and three (Q19, Q32, and Q36) were chosen by the expert panel independently of the statistical results. The choice of the 19 items kept in the final FRAGIRE grid was confirmed by IRT analysis (data not shown). The final 19 items of the final FRAGIRE grid demonstrated an excellent reproducibility with no statistically significant distribution of changes between day 0 and day 3 (Table 3). The structure of the final grid was supported by PCA (Fig. 2). Cronbach’s alpha was 0.69 (95%CI: 0.64–0.74), satisfying the consistency reliability (Table 4).

### Elaboration of a prognostic score

Of the final 19 FRAGIRE items, 16 were used for the prognostic score construction (For a detailed description see Additional file 5). Two items, Q34 and Q36, describing sexual dimension, were included in the construct with a view to future analysis, and one item, Q19 describing suicide dimension, given its non-neglected positive response rate was kept with public health screening in mind.

The “Set Test d’Isaacs” (STI) and the “Score de mémoire avec Indicage” (SMI) tests were maintained to assess the cognitive dimension (not included in prognostic score) and to provide complementary data for frailty evaluation (Additional files 6 and 7).

PCA, Cronbach alpha coefficient, and IRT results ensured an acceptable context for the prognostic score construction. PCAs conducted on the initial and final grids (Fig. 2) showed that the major part of the variance in data was explained by a first principal component (axis), which justified a unidimensional approach for the construction of frailty prognostic score. In fact, 18% and 6% of the variance in the 65-item grid was accounted for by the first two principal components, reflecting the importance of the first principal component.

**Fig. 3:**
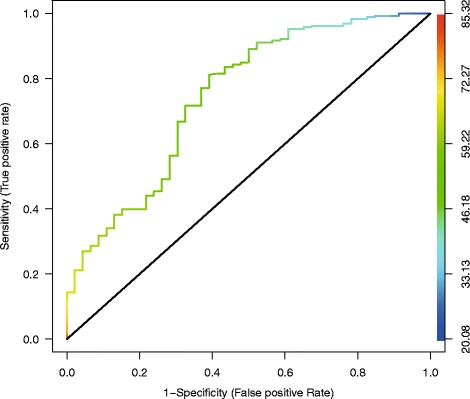
Receiver Operating Characteristic Curve for the prognostic score (AUC = 0.756)

In the final multivariate 19-item model (*N* = 339), six independent factors (Q5, Q24, Q30, Q31, Q32, and Q44) were found to be independently associated with “request help status” (*P* < .1) (Table 5). The model exhibited excellent discrimination ability (AUC = 0.85) and good calibration (Hosmer-Lemeshow *P* = 0.5800), reflecting an optimal agreement between prediction by the final model and actual observation. Bootstrapping results for internal validation reflected the robustness of the final model, especially for parameters significantly associated with “help requested status” (Table 5). The FRAGIRE prognostic score was normally distributed with a mean score of 55.7 (±10.5). In the FH group, the average score was significantly higher than in the NFH group (57.1 [±9.5] vs 46.4 [±12.1]; *P* < .0001). The score exhibited excellent discrimination ability (AUC 0.756) (Fig. 3). A score of 49.5 allowed efficiently and significantly discriminate individuals requesting for help from others (*P* < .0001), with sensitivity of 81%, specificity of 61%, positive predictive value of 93%, negative predictive value of 34%, and a global predictive value of 78%. When the elderly population is to be divided in three groups of interest (low, intermediate, and high probability of request help), FRAGIRE score tertiles (P33 = 52; P66 = 63) and the ROC curves discriminated between the groups with thresholds of 50 and 60.

**Table 5:** Univariate and multivariate unconditional logistic analyses on determinant and status of help beneficiary

Dimensions			Univariate analysis					Multivariate analysis				
								Full model / AUC = 0.7927				
								(*N* = 339)				
			Total	Helped	OR	95% CI	*P*-value	ß estimate	OR	95% CI	*P*-value	ß Internal validation (95% Bootstrap CI)
Global health status	Health status	Q1	379	332	0.66	0.54–0.80	< .0001	−0.048	0.95	0.69–1.32	0.7706	−0.38–0.26
	number of hospitalizations within the last 6 moths	Q4	380	333	1.14	0.62–2.09	.663	−0.083^a^	0.92	0.44–1.91	.823	−0.87–0.69
Psychological	General well-being	Q5	381	334	0.66	0.54–0.80	<.0001	−0.262	0.77	0.59–1.00	.051	−0.51–0.03
	Happiness	Q8	380	333	0.59	0.38–0.93	0.022	−0.084	0.92	0.51–1.66	.780	−0.76–0.86
	Tireness	Q16	383	336	1.67	1.18–2.40	.004	0.011	1.01	0.64–1.60	.961	−0.46–0.57
Environmental	Feeling of loneliness/abandonment	Q24	380	333	1.92	1.13–3.24	.015	0.541	1.72	0.93–3.18	.084	−0.18–1.41
	Sufficient financial resources	Q30	379	332	0.46	0.30–0.70	.0003	−0.568	0.57	0.34–0.94	.028	−1.20–0.00
Cultural	Use of internet	Q31	382	335	0.49	0.33–0.72	0.0003	−0.846	0.43	0.26–0.71	.0009	−1.36–-0.045
	Participation in activities	Q32	381	334	0.94	0.64–1.40	.772	0.433^a^	1.54	0.95–2.50	.078	−0.14–0.92
Sexual	Troubled by signs of aging	Q34	381	334	1.31	0.93–1.86	.125					
	Interest in sexual activity	Q36	376	330	0.73	0.42–1.28	.274					
Burden of help	Helping other relatives	Q37–38	380	333	0.81	0.63–1.04	.102	−0.076	0.93	0.69–1.25	.620	−0.39–0.36
Nutritional	Problems with taste	Q40	383	336	1.01	0.60–1.71	.958	−0.270	0.76	0.42–1.37	.368	−1.00–0.92
	Number of dental consultations	Q44	383	336	0.63	0.43 –0.92	.017	−0.462	0.63	0.40 – 1.0	.049	−0.96–0.14
Mobility	Falls	Q54	382	335	1.48	0.90 –2.44	.120	0.274	1.31	0.74–2.34	.351	−0.41–1.05
		Q56	368	322	1.71	1.16–2.53	.007	0.104	1.11	0.69–1.79	.672	−0.59–0.63
	Physical difficulties	Q55	380	333	1.54	1.16–2.05	.003	0.037	1.04	0.69–1.56	.856	−0.30–0.53
Section for examiner	Global health status	Q63	381	334	0.66	0.54–0.81	< .0001	−0.155	0.86	0.64–1.15	.301	−0.52–0.18

*CI* confidence interval

^a^ The ß estimated are not in the «expected» direction. For these estimations, a panel of experts decided to change the direction (positive to negative or negative to positive) without any changes to the value estimated for the contribution of these items in the score elaboration. All items were considered as ordinal categorical variables

**Fig. 4:**
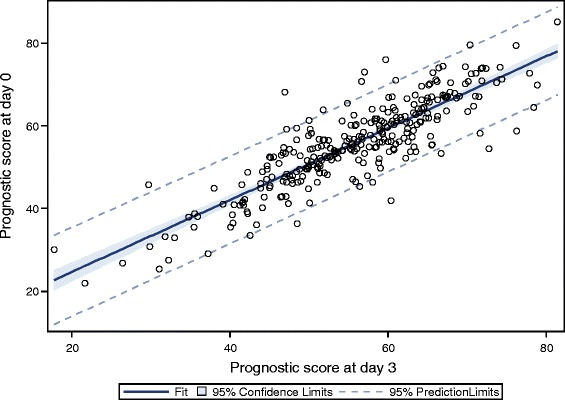
Linear regression between the individual prognostic score on day 0 and 3 (*N* = 293, *R* ^2^ = 0.74, *P* < .0001)

Linear regression and Pearson correlation analysis of the FRAGIRE prognostic scores between day 0 and day 3 (*N* = 293) showed an excellent correlation between the two measurements (*R* ^2^ = 0.74, *P* < 0.0001 and *R* ^2^ = 0.86, *P* < 0.0001, respectively, Fig. 4). Intraclass correlation coefficient scores were also excellent allowing a rejection of H0 (ICC > 0.86 for all methods, Table 6).

**Table 6:** Intraclass correlations for inter-rater reliability

Winer reliability: single score	Winer reliability: mean of k scores	Shrout-Fleiss reliability single score	Shrout-Fleiss reliability: random set	Shrout-Fleiss reliability: fixed set	Shrout-Fleiss reliability: mean k scores	Shrout-Fleiss rel: rand set mean k scores	Shrout-Fleiss rel: fixed set mean k scores
0.860	0.925	0.860	0.860	0.860	0.925	0.925	0.925

The FRAGIRE prognostic score significantly (*P* < .05) and negatively correlated with the MMSE global score and all dimensions of the SF-36, reflecting a satisfactory convergent validity (Table 7).

**Table 7:** Prognostic score correlation with the Mini Mental State Examination score and the SF-36 dimensions

	Number	Mean	SD	Median	Min.	Max	Pearson correlation analysis with the normalized prognostic score		
							N	Correlation coefficient	*P*-value
Normalized FRAGIRE score	293	55.7	10.5	55.8	22.0	85.1	293	1	
MMSE score on day 0	385	24.3	4.3	25.0	0	30.0	293	−0.13	0.028
SF-36									
Physical functioning	382	38.8	24.1	35.0	0	100	293	−0.465	< .0001
Role limitations--physical	381	39.4	39.4	25.0	0	100	293	−0.360	< .0001
Bodily pain^a^	379	46.3	22.1	45.0	0	100	292	−0.403	< .0001
Bodily pain -^b^	379	42.7	20.6	410	0	100	292	−0.390	< .0001
General health perceptions^a^	381	43.9	16.9	45.0	0	100	293	−0.520	< .0001
General health perceptions- ^b^	381	44.9	17.6	45.0	0	100	293	−0.532	< .0001
Emotional well-being	380	58.3	17.1	58.0	5.0	100	293	−0.482	< .0001
Role-emotional	376	53.9	44.6	66.7	0	100	289	−0.356	< .0001
Social functioning	379	72.2	22.7	75.0	0	100	292	−0.320	< .0001
Vitality	380	41.2	17.7	40.0	0	100	293	−0.530	< .0001

*MMSE* Mini Mental State Examination, *SF*-*36* Short Form-36 Health Survey

^a^ RAND scoring (RAND corporation)

^b^ NEMC scoring (New England Medical Center)

## Discussion

This paper describes the development and validation of a new frailty-specific instrument, the Frailty GIR Evaluation (FRAGIRE) consisting of 19 clinically relevant health or environmental items based on literature review and expert recommendations. The instrument showed good discriminative capability, sensitivity and specificity as reflected by the AUC analysis, good reliability with the Hosmer Lemeshow assessment of the calibration,, and excellent construct convergent validity with the strong correlation between the score and MMSE and SF-36 results. The Cronbach's alpha for the developed tool scored as high as 0.69. with a 95% bootstrap confidence interval equal to (0.64–0.73,) was considered as an acceptable result for this analysis as the 0.7 value was included in the confidence interval. This analysis demonstrated that the FRAGIRE instrument is clinically sensible and discriminates between groups of elderly.

The originality of our research was to provide a multidimensional tool to measure frailty and produce new simple prognostic score based on selected items and dimensions to identify high-risk frail older subjects. The great advantage of the tool is its easy implementation by a public health social worker without formal training in geriatric care. Noticeably, the final FRAGIRE tool showed an agreement for all selected items recorded on day 0 and day 3, highlighting an excellent reproducibility of these items.

Di Bari et al. recently developed and tested a 10-item screening questionnaire to intercept frailty in large cohort of older community-dwelling individuals.^5^ Compared with this Italian model, the 19-item FRAGIRE grid has advantages because it includes emotional and environmental aspects in addition to functional status, and seems to present a better discriminatory ability, has been rigorously tested for repeatability and convergent validity, and assesses multiple domains.

Each item in the final FRAGIRE tool was included as clinically necessary and relevant. Self-assessment of frailty by the individuals themselves (in the global health status dimension), a measure that provides an idea of its positioning compared to non-frail people of similar age, appeared to be a good component of initial assessment with good discrimination ability and an acceptable contribution to principal components in the PCA analysis. Hospitalization, the deciding factor in the functional ability of the frail elderly [31], likewise showed these properties. Three items in the psychological dimension, general well-being, happiness, and tiredness, were also retained in the final tool due to their clinical relevance that is close association with frailty [32]. We considered that these items would prompt the dynamism of the structure. Our *a priori* choice strategy was confirmed by statistical analyses showing that this structure had good discrimination ability and an acceptable contribution for all those items. In the environmental dimension, feeling of loneliness and/or abandonment and financial situation level were kept in the final FRAGIRE grip as these appeared the most relevant in terms of discrimination ability. These social factors, including isolation and financial situation, have been shown to be involved in the vulnerability process [33]. Despite a low internal consistency (Cronbach’s coefficient of < 0.50), two items in the socio-cultural dimension, use of Internet and participation to group activities, were maintained in the final grid due to their high discrimination abilities and contribution to PCA and due to clinical relevance recognized by the expert group, respectively. The structure incorporating these characteristics may be more successful in targeting social isolation and adaptability in older people. Four other variables, responsibility towards relatives (burden of help dimension), the number of falls within the last 6 months, physical difficulties, and walking speed (mobility dimension) were also retained as relevant in the final FRAGIRE tool as these attest to the dynamism, the non-sedentary and the non-social isolation of assessed persons [23], or showed high discrimination ability and contribution in PCA correlation. The three mobility items were shown to be strongly associated with frailty.^1^

Although some items were not included in the final score, these were retained due to their importance from a public health perspective. For instance, the FRAGIRE scale contains a suicide item that can be highly relevant in the assessment of the elderly. Suicide is specifically of concern in older adults as suicide rates increase with advanced age. However despite its potential as risk factor, suicide in the elderly people still receives little focus in terms of specific preventive strategies or research. Our analysis showed that suicide ideas were more frequent in our population (8%) than in the general population according to the 2010 Health Barometer in France (3.9%) [34], which emphasizes the importance of detection of the suicide risk in the elderly population. Even if our data do not show statistically significant correlation with frailty, we believe that the collection of this information for suicide prevention policies is of interest. Along the same line of though, the cognitive dimension with MIS-IST pairing was retained in the final model. The MIS-IST pairing is quick and simple to score and the efficacy of the MIS and IST combination in predicting short-term development of dementia in a group of people with questionable dementia has been previously reported.^20^ Although positive results cannot be used to definitely diagnose dementia, it can be considered a useful screening procedure for all types of dementia and can be a good way of directing the elderly people towards specialized consultation. We hope that this approach in the FRAGIRE grid will help to develop specific detection and prevention strategies.

Our study has some limitations that should be noted. First, our study did not consider socioeconomic status parameter that could provide important information about health status including frailty. Indeed, we hypothesized that the elderly from GIR 5 and 6 population who claim PAP will be potentially more at risk to become frail than those who do not. Whatever the amount of the retirement pension received, the elderly people could be eligible for the financial help weighted according to the pension received. By definition, all socioeconomic status measures can be found in each group, but we cannot guarantee their balance between the two populations.

The FRAGIRE grid was developed to be enunciated to the elderly population (corresponding to a hetero-assessment). While this method seems to be more adapted to elderly population than a self-reported questionnaire regarding the targeted population and to the tests included in the grid, it can raise the issue of the inter-rater reliability for the examiner dimension. The inter-rater reliability of examiners’ judgement however could not be assessed in our study because the assessment was made by only one social worker per elderly.

Another potential limitations of our study are the difficulty encountered for NFH enrollment and that we did not compare the FRAGIRE grid with frailty measures such as the Fried and Rockwood methods. In order to prevent excessive burden in data collection by social and other healthcare workers such very time-consuming and laborious process was considered unessential at this time of the development process of the FRAGIRE tool. However, future studies could potentially address this issue.

Further, this study involves a cross-sectional design. Our findings suggest that the FRAGIRE grid should now be validated prospectively to ensure that the score could predict frailty and thus help to make decision on resources allocation. The FRAGIRE tool is currently in use in France and is being tested in a prospective external validation cohort for sensitivity to change, for reproducibility to improve the proposed prognostic score, and for more accurate determination the cutoff threshold of the FRAGIRE score. The primary objective of the external validation is to assess the discriminative ability of the FRAGIRE grid for predicting the loss of autonomy; an indicator of frailty, i.e. the tilting of the elderly people to a GIR of 4 or lower from GIR 5 and 6 elderly subjects. Thus, the conduct of elderly frailty assessment will be performed in an accurate and objective way without taking into account hypothesis of the NFH and FH groups‘ frailty surrogacy. Secondary objective that include, the assessment of the status FH and NFH groups frailty surrogacy to validate the hypothesis involved in the present study. However, the internal-validation ensures a reliable estimate of performance for subjects similar to those of the present development sample. Another limitation is that the FRAGIRE score can only be estimated if all items and tests are answered. It would be important to perform a missing data sensitivity analysis on the prospective validation cohort with the items selected in the final FRAGIRE grid to assess their potential association with frailty status observed and to propose, if an association is highlighted, an alternative in the determination of the prognostic score.

## Conclusion

In summary, the FRAGIRE grid and derived instruments have been constructed in response to a lack of any validated tool for frailty screening in the GIR 5 and 6 French population. It appears to be a potential reliable and effective tool for identifying elderly individuals at risk to become frail by a public health social worker without formal training in geriatric care and for providing a simple prognostic score for frailty prediction.

## Additional files

Additional file 1: English and French version of the AGGIR grid. (DOCX 112 kb)
Additional file 2: Definition of the eligibility criteria in the financially and non-financially helped group of subjects enrolled in the study. (DOCX 13 kb)
Additional file 3: The FRAGIRE “pre-grid” (A) English version and (B) Original version in French. (DOCX 65 kb)
Additional file 4: The final FRAGIRE grid (A) English version and (B) Original version in French. (DOCX 45 kb)
Additional file 5: Details on the construction of the prognostic score. (DOCX 46 kb)
Additional file 6:Table S1. Description of the Memory Impairment Screen results in the overall population and according to the financially helped status at day 0 and day 3. (DOCX 15 kb)
Additional file 7: Table S2. Description of the Isaacs Set Test results in the overall population and according to the financially helped status. (DOCX 15 kb)

## Abbreviations

AUC: Area under the curve
CCMSA: Caisse Centrale de la Mutualité Sociale Agricole
CDT: Clock-drawing test
CETAF: Centre Technique d'Appui et de Formation des Centre d'Examen de Santé
CI: Confidence interval
CNAV: Caisse Nationale d'Assurance Vieillesse
FH: Financially-helped
FRAGIRE: Frailty Groupe Iso-Ressource Evaluation
GIR: Groupe Iso-Ressource
ICC: Intraclass correlation coefficient
IRT: Item response theory
IST: Isaacs Set Test
MIS: Memory Impairment Screen
MMSE: Mini Mental State Examination
NFH: Non-financially helped group
PAP: Pension additional plan
PCA: Principal component analysis
ROC: Receiver operating characteristic
SEGAm: Short Emergency Geriatric Assessment
SF-36: Medical Outcome Study Short Form-36
SHARE: Survey of Health, Aging and Retirement in Europe
