# Erratum to: Development and validation of the FRAGIRE tool for assessment an older person’s risk for frailty

**DOI:** 10.1186/s12877-016-0385-0

**Published:** 2016-12-09

**Authors:** Dewi Vernerey, Amelie Anota, Pierre Vandel, Sophie Paget-Bailly, Michele Dion, Vanessa Bailly, Marie Bonin, Astrid Pozet, Audrey Foubert, Magdalena Benetkiewicz, Patrick Manckoundia, Franck Bonnetain

**Affiliations:** 1 Methodological and Quality of Life in Oncology Unit, INSERM U1098, University Hospital of Besançon, Besançon, France; 2 National clinical research Platform for Quality of life in Oncology, Besançon, France; 3 Department of psychiatry, EA 481, University Hospital of Besançon, Besançon, France; 4 Centre Georges Chevrier «Knowledge: norms and sensitivities», UMR CNRS 7366, University of Burgundy, Dijon, France; 5 Interregional Gerontology Pole from Burgundy and Franche-Comté, Dijon, France; 6 GERCOR, Groupe Coopérateur Multidisciplinaire en Oncologie, Paris, France; 7 Department of Geriatrics and Internal Medicine, Hospital of Champmaillot, University Hospital, Dijon, France; 8 Inserm/U1093 Cognition, Action and Sensorimotor Plasticity, University of Burgundy Franche-Comté, Dijon, France

## Erratum

It has come to our attention that there is an error in one of the author names for this article [1]. The author name Patrick Manckoundia was incorrectly spelt as Patrick Mankoundia. The original article has now been updated.
