# Supplementary material for: Development and validation of the FRAGIRE tool for assessment an older person’s risk for frailty
Source: BMC Geriatr. 2016 Nov 17;16:187. doi: 10.1186/s12877-016-0360-9 (PMC5114762; doi:10.1186/s12877-016-0360-9)

**English and French version of the AGGIR grid**

English version

GIR 1: bedridden or confined to an armchair and mental faculties severely impaired

GIR 2: confined or impaired mental faculties

GIR 3: help several times a day for activities of daily life

GIR 4: loss of autonomy for transferring, sometimes also regarding toileting or dressing, or mobile but needs to perform activities of daily life, including eating

GIR 5: help for bathing and home care

GIR 6: autonomous

French version


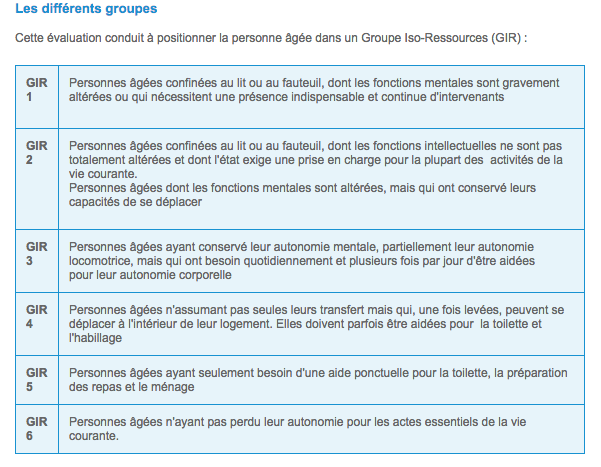

Supplement: Additional file 1: — English and French version of the AGGIR grid. (DOCX 112 kb) [file 12877_2016_360_MOESM1_ESM.docx]
