# Supplementary material for: Development and validation of the FRAGIRE tool for assessment an older person’s risk for frailty
Source: BMC Geriatr. 2016 Nov 17;16:187. doi: 10.1186/s12877-016-0360-9 (PMC5114762; doi:10.1186/s12877-016-0360-9)
Supplement: Additional file 2: — Definition of the eligibility criteria in the financially and non-financially helped group of subjects enrolled in the study. (DOCX 13 kb) [file 12877_2016_360_MOESM2_ESM.docx]

**Definition of the eligibility criteria in the financially and non-financially helped group of subjects enrolled in the study.**

The study sample of 385 older adults (initially) consisted of:

**Population 1:** 338 financially helped group of subjects:

Including those:

- Who are helped, received pension additional plan (PAP) benefit for the first time, those who received benefits for 1 year, those whose PAP has just expired; those who are requesting a PAP revision;

- Who are receiving a pension under the general, agricultural, or independent regimen (minimum age of 60 or 65 years or irrespective of age depending on the site);

- Who are living at home in Bourgogne and Franche-Comté;

- Who have GIR score of 5 or 6 (degree of autonomy) on the basis of the Autonomie Gérontologie Groupes Iso-Ressources (AGGIR) scale;

- Who are receiving retirement benefits of Caisse d’Assurance Retraite et de Santé Au Travail Bourgogne Franche-Comté (CARSAT BFC), Mutualité Sociale Agricole Franche-Comté (MSA FC), Caisse Régionale de la Mutualité Sociale Bourgogne (CRMSAB), or Régime Social Indépendant Franche-Comté (RSI FC);

- With the capacity to answer the questions of the proposed grid;

- With signed and dated information form and no opposition.

**Population 2:** 47 Non-financially helped group of subjects
