# Supplementary material for: Development and validation of the FRAGIRE tool for assessment an older person’s risk for frailty
Source: BMC Geriatr. 2016 Nov 17;16:187. doi: 10.1186/s12877-016-0360-9 (PMC5114762; doi:10.1186/s12877-016-0360-9)
Supplement: Additional file 3: — The FRAGIRE “pre-grid” (A) English version and (B) Original version in French. (DOCX 65 kb) [file 12877_2016_360_MOESM3_ESM.docx]

The FRAGIRE “pre-grid” (A) English version and (B) Original version in French

1. English version

**Dimension General Health status –psychological**

**Please mark the number (from 0 to 10) that best reflects your answer to the following questions.**

**Each question refers to how you felt about your health in general the last week and how you feel about your health today**

**How do you describe**:

**1-** Your health status? **(Q1)**

0 1 2 3 4 5 6 7 8 9 10

(0 the worst health you can imagine- 10 the best health you can imagine)

**2-** Your health status compared to people of the same age as you? **(Q2)**

0 1 2 3 4 5 6 7 8 9 10

(0 the worst health you can imagine- - 10 the best health you can imagine)

**Circle the best answer from those proposed:**

**3-** At the present time you take more than 5 medications per day? **(Q3)**

Yes / No / I don't know

**4-** How many times were you hospitalized within the last 6 months? **(Q4)**

0 / 1-2 times / More than 2 / I don't know

**Dimension Psychological– depression, anxiety, difficulty sleeping, enjoyment, suicide ideation**

**Please mark the number (from 0 to 10) that best reflects your answer to the following questions.**

**Each question refers to how you felt about your health in general the last week and how you feel about your health today**

**How would you describe**:

**5-** Your general well-being **(Q5)**

0 1 2 3 4 5 6 7 8 9 10

(0 the worst health you can imagine- 10 the best health you can imagine)

**6-** Your mood **(Q6)**

0 1 2 3 4 5 6 7 8 9 10

(0 the worst health you can imagine- 10 the best health you can imagine)

**Circle the best answer from those proposed**

**In the last months:**

**7-** Have you been unhappy and depressed? **(Q7)**

1 Not at all / 2 A little/ 3 Quite a bit/ 4 Very much

**8-** Have you been happy most of the time? **(Q8)**

1 Not at all / 2 A little/ 3 Quite a bit/ 4 Very much

**9-** Has your felt that your life is enjoyable? (Q9)

1 Not at all / 2 A little/ 3 Quite a bit/ 4 Very much

**10-** Have you often felt discouraged and sad? (Q10)

1 Not at all / 2 A little/ 3 Quite a bit/ 4 Very much

**11-** Have you kept a generally positive and optimistic outlook on life? (Q11)

1 Not at all / 2 A little/ 3 Quite a bit/ 4 Very much

**12-** Have you felt that you are playing a useful role in life ? (Q12)

1 Not at all / 2 A little/ 3 Quite a bit/ 4 Very much

**13-** Have you felt motivated to continue your usual activities and leisure? **(Q13)**

1 Not at all / 2 A little/ 3 Quite a bit/ 4 Very much

**14-** Have you often felt tensed, angry, stressed **(Q14)**

1 Not at all / 2 A little/ 3 Quite a bit/ 4 Very much

**15-** Have you had difficulties sleeping? **(Q15)**

1 Not at all / 2 A little/ 3 Quite a bit/ 4 Very much

**16-** Have you been tired during the day? **(Q16)**

1 Not at all / 2 A little/ 3 Quite a bit/ 4 Very much

**17-** Have you been capable of enjoying your daily activities? **(Q17)**

1 Not at all / 2 A little/ 3 Quite a bit/ 4 Very much

**18-** Did you continue to consider life in a positive way? **(Q18)**

1 Not at all / 2 A little/ 3 Quite a bit/ 4 Very much

**19-** Have you suffered so much that you start having suicide ideation? **(Q19)**

1 Not at all / 2 A little/ 3 Quite a bit/ 4 Very much

**Dimension COGNITIVE – memory, praxis**

**Circle the best answer from those proposed.**

**Lately:**

**20-** Have you had difficulties to concentrate on your daily activities (for example, reading newspaper, watching TV)? **(Q20)**

1 Not at all / 2 A little/ 3 Quite a bit/ 4 Very much

**21-** Have you had difficulties in remembering things? **(Q21)**

1 Not at all / 2 A little/ 3 Quite a bit/ 4 Very much

**TESTING PHASE:**

**Perform the rapid diagnostic tests**: the Mini Mental State Examination (MMSE), the clock-drawing test (CDT), the serious mental illnesses (SMI) test, and Isaac set test (STI).

Perform the Bi-Digital O-Ring Test (BDORT): a diagnostician employs the thumb and forefinger of each hand, formed in the shape of an O, to attempt to force apart an O shape formed by the patient who places the fingertips of their thumb and one of their remaining fingers together.

**Dimension ENVIRONMENTAL – isolation,** societal **and** family breakdown**…**

22- One or more people have supported you? **(Q22)**

Yes/ No/ I don’t know

**Circle the best answer from those proposed.**

**In the last weeks:**

23- Have you been satisfied with support you got? **(Q23)**

1 Not at all / 2 A little/ 3 Quite a bit/ 4 Very much

24- Have you felt lonely or abandonment? **(Q24)**

1 Not at all / 2 A little/ 3 Quite a bit/ 4 Very much

25- Have you felt that your relationship with other people deteriorated? **(Q25)**

1 Not at all / 2 A little/ 3 Quite a bit/ 4 Very much

26- Have been there an event that has changed your life? **(Q26)**

(admission to clinical center, hospitalization, divorce, death of a loved one, loss of an animal companion)

Yes / No/ I don’t know

27- Have you felt like going outside your place? **(Q27)**

Yes / No/ I don’t know

28- Have you had means of getting around? **(Q28)**

Yes / No/ I don’t know

29- Your current situation has caused you financial difficulties? **(Q29)**

1 Not at all / 2 A little/ 3 Quite a bit/ 4 Very much

30- Your financial situation seemed sufficient to meet your needs? **(Q30)**

1 Not at all / 2 A little/ 3 Quite a bit/ 4 Very much

**Dimension CULTURAL – leisure, activities**

**Circle the best answer from those proposed.**

31- Do you use Internet? **(Q31)**

1 Not at all / 2 A little/ 3 Quite a bit/ 4 Very much

32- Do you participate in sport activities (sport, art, etc.) ? **(Q32)**

1 Not at all / 2 A little/ 3 Quite a bit

**Dimension SEXUAL**

**Circle the best answer from those proposed.**

33- Are you troubled by signs of weakening? **(Q33)**

1 Not at all / 2 A little/ 3 Quite a bit/ 4 Very much

34- Are you troubled by signs of aging?? **(Q34)**

1 Not at all / 2 A little/ 3 Quite a bit/ 4 Very much

35- Do you have positive self-image? **(Q35)**

1 Not at all / 2 A little/ 3 Quite a bit/ 4 Very much

36- Are you interested in sexual activity? **(Q36)**

1 Not at all / 2 A little/ 3 Quite a bit/ 4 Very much

**Dimension Burden of help – ROLE AIDANTS**

**Circle the best answer from those proposed**

**In the last weeks:**

37- Have you needed to provide support or assistance to your relatives? **(Q37)**

1 Not at all / 2 A little/ 3 Quite a bit/ 4 Very much

38- Have you felt responsible for your relatives? **(Q38)**

1 Not at all / 2 A little/ 3 Quite a bit/ 4 Very much

39- Have you found difficult to take care of yourself? **(Q39)**

1 Not at all / 2 A little/ 3 Quite a bit/ 4 Very much / 5 Don’t concern

**Dimension NUTRITIONAL – taste, appetite, denture**

**Circle the best answer from those proposed**

**In the last weeks**

40- Have you had problem with taste? **(Q40)**

1 Not at all / 2 A little/ 3 Quite a bit/ 4 Very much

41- Have you experienced lack of appetite? **(Q41)**

1 Not at all / 2 A little/ 3 Quite a bit/ 4 Very much

42- Have your food intake reduced in the last 6 months? **(Q42)**

1 Not at all / 2 A little/ 3 Quite a bit/ 4 Very much

43- Have you noticed weight loss within the last 6 months or that your clothes become too big/got larger in the last months? **(Q43)**

1 Not at all / 2 A little/ 3 Quite a bit/ 4 Very much

44- How many dental consultations have you had? **(Q44)**

0 / 1 / more than 1 / I don’t know

45- How often have you had dental pain **(Q45)**

Yes / No

46- Have you been able to eat on your own? **(Q46)**

Yes/ No / I don’t know

47- Have you had denture? **(Q47)**

Yes/ No

**Dimension NEUROSENSORY – vision, hearing**

**Circle the best answer from those proposed**

48- Have you experienced deterioration in vision in the last 6 months? **(Q48)**

1 Not at all / 2 A little/ 3 Quite a bit/ 4 Very much

49- Have you purchased glasses or you have changed yours in the last 6 months? **(Q49)**

Yes/ No /I don’t know

50- Have you find it hard to follow conversation due to a hearing problem in the last 6 months? **(Q50)**

1 Not at all / 2 A little/ 3 Quite a bit/ 4 Very much

51- Do you wear a hearing aid? **(Q51)**

Yes/ No

52- If yes, is it well adapted? **(Q52)**

Yes/ No / I don’t know

53- If not, have your hearing has deteriorated recently? **(Q53)**

Yes/ No /I don’t know

**Dimension Mobility – balance, walk, usual movements**

**Circle the best answer from those proposed**

54- Have you fallen in the last 6 months? **(Q54)**

0 / 1 / more than once / I don’t know

**In the last weeks:**

55- Have you had difficulties doing some physical activities such as carrying a loaded shopping bag or suitcase? **(Q55)**

1 Not at all / 2 A little/ 3 Quite a bit/ 4 Very much

**Test:** the 4-meter walk test with the cut-off point for 3 meters: **(Q56)**

normal ≥ 1 m/s

impairment of balance and walking: between 0.65 and < 1 m/s

deficiency (global) : < 0.65 m/s

57- Have you washed yourself? **(Q57)**

Yes/ No /I don’t know

58- If not, have you had any help? **(Q58)**

Yes/ No /I don’t know

59- Have you had difficulties doing shopping on your own? **(Q59)**

Yes/ No / I don’t know

60- If yes, have you received any help? **(Q60)**

Yes/ No /I don’t know

61- Have you done cleaning on your own? **(Q61)**

Yes/ No /I don’t know

62- If not, have you received any help? **(Q62)**

Yes/ No /I don’t know

**Section for examiner**

**Please mark the number (from 0 to 10) that best reflects your answer to the following questions.**

**Each question refers to how you felt about the older patient you have evaluated**

**How would you describe**:

63- Her/his glabal heatlh status? **(Q63)**

0 1 2 3 4 5 6 7 8 9 10

(0 -the worst health you can imagine,10 - the best health you can imagine)

64- Her/his health status compared to people of the same age? **(Q64)**

0 1 2 3 4 5 6 7 8 9 10

(0 -the worst health you can imagine,10 - the best health you can imagine)

65- In the light of this questionnaire, do you think that there is a risk of deterioration of her/his health status during the next year? **(Q65)**

0 1 2 3 4 5 6 7 8 9 10

(0 - Very big chance, 10- very unlikely)

1. Original version in French

**Dimension ETAT DE SANTE GLOBAL – ressenti physiologique**

**Veuillez cocher le chiffre (de 0 à 10) qui reflète le mieux votre réponse à la question suivante.**

**Cette question porte sur ce que vous avez ressenti au cours de la semaine qui vient de s’écouler, aujourd’hui compris.**

Comment décririez-vous :

1- votre état de santé ? **(Q1)**

0 1 2 3 4 5 6 7 8 9 10

(0 Aussi mauvais que possible - 10 Aussi bon que possible)

2- votre état de santé par rapport aux personnes de votre âge ? **(Q2)**

0 1 2 3 4 5 6 7 8 9 10

(0 Aussi mauvais que possible - 10 Aussi bon que possible)

**Entourez la réponse qui convient le mieux parmi celles proposées**

3- A l’heure actuelle prenez-vous plus de 5 médicaments différents par jour ? **(Q3)**

Oui / Non / Ne sait pas

4- Combien de fois avez-vous été hospitalisé(e) au cours des 6 derniers mois ? **(Q4)**

0 / 1 à 2 fois / Plus de 2 fois / Ne sait pas

**Dimension PSYCHIQUE – dépression, anxiété, sommeil, plaisir, suicide**

**Veuillez cocher le chiffre (de 0 à 10) qui reflète le mieux votre réponse à la question suivante.**

**Cette question porte sur ce que vous avez ressenti au cours de la semaine qui vient de s’écouler, aujourd’hui compris.**

Comment décririez-vous :

5- votre bien-être général ? **(Q5)**

0 1 2 3 4 5 6 7 8 9 10

(0 Aussi mauvais que possible - 10 Aussi bon que possible)

6- votre moral ? **(Q6)**

0 1 2 3 4 5 6 7 8 9 10

(0 Aussi mauvais que possible - 10 Aussi bon que possible)

**Entourez la réponse qui vous convient le mieux parmi les celles proposées**

**Au cours du dernier mois :**

7- Avez-vous été malheureux (se) et déprimé(e) ? **(Q7)**

1 Pas du tout / 2 Un peu / 3 Assez / 4 Beaucoup

8- Etes-vous heureux (se) la plupart du temps ? **(Q8)**

1 Pas du tout / 2 Un peu / 3 Assez / 4 Beaucoup

9- Avez-vous le sentiment que votre vie n'est pas assez remplie ? **(Q9)**

1 Pas du tout / 2 Un peu / 3 Assez / 4 Beaucoup

10- Vous sentez-vous souvent découragé(e) et triste ? **(Q10)**

1 Pas du tout / 2 Un peu / 3 Assez / 4 Beaucoup

11- Avez-vous continué à considérer la vie de manière positive ? **(Q11)**

1 Pas du tout / 2 Un peu / 3 Assez / 4 Beaucoup

12- Avez-vous eu le sentiment de jouer un rôle utile dans la vie ? **(Q12)**

1 Pas du tout / 2 Un peu / 3 Assez / 4 Beaucoup

13- Vous êtes-vous senti(e) motivé(e) à poursuivre vos loisirs et activités habituels ? **(Q13)**

1 Pas du tout / 2 Un peu / 3 Assez / 4 Beaucoup

14- Vous êtes-vous senti(e) fréquemment tendu(e), énervé(e) ou « stressé(e) »? **(Q14)**

1 Pas du tout / 2 Un peu / 3 Assez / 4 Beaucoup

15- Avez-vous eu des difficultés pour dormir ? **(Q15)**

1 Pas du tout / 2 Un peu / 3 Assez / 4 Beaucoup

16- Vous sentez-vous fatigué(e) pendant la journée ? **(Q16)**

1 Pas du tout / 2 Un peu / 3 Assez / 4 Beaucoup

17- Avez-vous été capable d’avoir du plaisir dans vos activités quotidiennes ? **(Q17)**

1 Pas du tout / 2 Un peu / 3 Assez / 4 Beaucoup

18- Avez-vous continué à considérer la vie de manière positive ? **(Q18)**

1 Pas du tout / 2 Un peu / 3 Assez / 4 Beaucoup

19- Souffrez-vous au point d’avoir des idées de suicide ? **(Q19)**

1 Pas du tout / 2 Un peu / 3 Assez / 4 Beaucoup

**Dimension COGNITIVE – mémoire, praxie**

**Entourez la réponse qui vous convient le mieux parmi les celles proposées**

**Ces derniers temps :**

**Entourez la réponse qui vous convient le mieux parmi les celles proposées**

**Ces derniers temps :**

20- Avez-vous eu des difficultés à vous concentrer sur certaines choses (par exemple pour lire le journal, regarder la télévision ou autre chose) ? **(Q20)**

1 Pas du tout / 2 Un peu / 3 Assez / 4 Beaucoup

21- Avez-vous eu des difficultés pour vous souvenir de certaines choses ? **(Q21)**

1 Pas du tout / 2 Un peu / 3 Assez / 4 Beaucoup

**PHASE DE TESTS :**

**Faire passer le test rapide de détection :** MMSE / Test de l'horloge / Score de Mémoire avec indiçage (SMI) / Set test d'Isaac (STI)

**Faire passer le test des anneaux :** exécution de 2 anneaux avec le pouce et l'index de chaque main emboîtés

**Dimension ENVIRONNEMENTALE – isolement, rupture sociale, familiale…**

22- Une ou plusieurs personnes s'occupent-elles de vous ou vous soutiennent ?   **(Q22)**

Oui / Non / Ne sait pas

**Entourez la réponse qui vous convient le mieux parmi celles proposées**

**Ces dernières semaines :**

23- Etes-vous satisfait(e) du soutien apporté par cette/ces personne(s) ? **(Q23)**

1 Pas du tout / 2 Un peu / 3 Assez / 4 Beaucoup

24- Avez-vous un sentiment de solitude et/ou d’abandon ? **(Q24)**

1 Pas du tout / 2 Un peu / 3 Assez / 4 Beaucoup

25- Estimez-vous que votre relation avec les autres s’est détériorée? **(Q25)**

1 Pas du tout / 2 Un peu / 3 Assez / 4 Beaucoup

26- Y-a-t-il eu un évènement marquant qui vous a affecté(e) dans votre vie récemment ?  **(Q26)**

(Entrée en institution, hospitalisation, divorce, décès d’un proche, d’un animal de compagnie)

Oui / Non / Ne sait pas

27- Avez-vous envie de sortir de chez vous ? **(Q27)**

Oui / Non / Ne sait pas

28- Avez-vous des solutions pour vous rendre là où vous en avez besoin hors de votre domicile ? **(Q28)**

Oui / Non / Ne sait pas

29- Votre situation actuelle vous cause t-elle des problèmes financiers ? **(Q29)**

1 Pas du tout / 2 Un peu / 3 Assez / 4 Beaucoup

30- Votre niveau de ressources vous semble-t-il suffisant ? **(Q30)**

1 Pas du tout / 2 Un peu / 3 Assez / 4 Beaucoup

**Dimension CULTURELLE – loisirs, occupation**

**Entourez la réponse qui vous convient le mieux parmi celles proposées**

31- Utilisez-vous Internet ? **(Q31)**

1 Pas du tout / 2 Un peu / 3 Assez / 4 Beaucoup

32- Participez-vous à des activités (clubs sportif, artistique…) ? **(Q32)**

1 Pas du tout / 2 Un peu / 3 Assez

**Dimension SEXUELLE**

**Entourez la réponse qui vous convient le mieux parmi celles proposées**

33- Etes-vous affecté(e) par des signes d’affaiblissement de votre corps ? **(Q33)**

1 Pas du tout / 2 Un peu / 3 Assez / 4 Beaucoup

34- Etes-vous affecté(e) par des signes visibles du vieillissement ? **(Q34)**

1 Pas du tout / 2 Un peu / 3 Assez / 4 Beaucoup

35- Avez-vous une image positive de vous ? **(Q35)**

1 Pas du tout / 2 Un peu / 3 Assez / 4 Beaucoup

36- Vous intéressez-vous à la sexualité ? **(Q36)**

1 Pas du tout / 2 Un peu / 3 Assez / 4 Beaucoup

**Dimension FARDEAU – ROLE AIDANTS**

**Entourez la réponse qui vous convient le mieux parmi celles proposées**

**Ces dernières semaines :**

37- Vous arrive t-il de devoir vous occuper d’un proche ? **(Q37)**

1 Pas du tout / 2 Un peu / 3 Assez / 4 Beaucoup

38- Vous sentez-vous responsable de ce proche ? **(Q38)**

1 Pas du tout / 2 Un peu / 3 Assez / 4 Beaucoup

39- Trouvez-vous pénible/difficile de vous occuper de votre entourage ?  **(Q39)**

1 Pas du tout / 2 Un peu / 3 Assez / 4 Beaucoup / 5 Non concerné

**Dimension NUTRITIONNELLE – gout, appétit, bucco-dentaire**

**Entourez la réponse qui vous convient le mieux parmi celles proposées**

**Ces dernières semaines :**

40- Avez-vous des difficultés pour reconnaître le goût des aliments que vous consommez ?  **(Q40)**

1 Pas du tout / 2 Un peu / 3 Assez / 4 Beaucoup

41- Avez-vous manqué d'appétit ? **(Q41)**

1 Pas du tout / 2 Un peu / 3 Assez / 4 Beaucoup

42- Avez-vous réduit la quantité de vos repas durant les 6 derniers mois ? **(Q42)**

1 Pas du tout / 2 Un peu / 3 Assez / 4 Beaucoup

43- Avez-vous involontairement perdu du poids durant ces 6 derniers mois ou avez-vous remarqué que vos vêtements sont devenus trop larges/trop grands au cours de ces derniers mois ? **(Q43)**

1 Pas du tout / 2 Un peu / 3 Assez / 4 Beaucoup

44- De combien de consultations dentaires annuelles avez-vous bénéficié? **(Q44)**

0 / 1 / plus de 1 / Ne sait pas

45- Avez-vous fréquemment des douleurs dentaires?  **(Q45)**

Oui / Non

46- Etes-vous capable de vous alimenter correctement par la bouche sans aide ? **(Q46)**

Oui / Non / Ne sait pas

47- Avez-vous un dentier ? **(Q47)**

Oui / Non

**Dimension NEUROSENSORIELLE – vue, audition**

**Entourez la réponse qui vous convient le mieux parmi celles proposées**

48- Avez-vous l’impression que votre vue s’est dégradée durant ces 6 derniers mois ? **(Q48)**

1 Pas du tout / 2 Un peu / 3 Assez / 4 Beaucoup

49- Avez-vous acquis des lunettes ou changé les vôtres au cours des 6 derniers mois ? **(Q49)**

Oui / Non / Ne sait pas

50- Avez-vous l’impression de moins bien suivre les conversations en raison d’une gêne auditive depuis 6 mois ?  **(Q50)**

1 Pas du tout / 2 Un peu / 3 Assez / 4 Beaucoup

51- Portez-vous un appareil auditif ? **(Q51)**

 Oui / Non

52- Si oui, est-il bien adapté à votre audition ? **(Q52)**

Oui / Non / Ne sait pas

53- Si non, votre audition s’est-elle dégradée ces derniers temps ? **(Q53)**

Oui / Non / Ne sait pas

**Dimension MOTRICITE – équilibre, marche, gestes usuels**

**Entourez la réponse qui vous convient le mieux parmi celles proposées**

54- Avez-vous chuté au cours des 6 derniers mois ? **(Q54)**

0 / 1 / plus de 1 / Ne sait pas

**Ces dernières semaines :**

55- Avez-vous des difficultés à faire certains efforts physiques pénibles comme porter un sac à provision chargé ou une valise ? **(Q55)**

1 Pas du tout / 2 Un peu / 3 Assez / 4 Beaucoup

**Test :** vitesse de marche sur 4 m avec 3 seuils : **(Q56)**

normale ≥ 1 m/s

altération de l'équilibre et de la marche : entre 0,65 et < 1 m/s

fragilité (globale) : < 0,65 m/s

57- Faites-vous votre toilette seul(e) ? **(Q57)**

Oui / Non / Ne sait pas

58- Si non, avez-vous une aide ? **(Q58)**

Oui / Non / Ne sait pas

59- Eprouvez-vous des difficultés à faire vos courses seul(e) ? **(Q59)**

Oui / Non / Ne sait pas

60- Si oui, avez-vous une aide ? **(Q60)**

Oui / Non / Ne sait pas

61- Faites-vous le ménage seul(e) ? **(Q61)**

Oui / Non / Ne sait pas

62- Si non, avez-vous une aide ? **(Q62)**

Oui / Non / Ne sait pas

**PARTIE EVALUATEUR**

**Veuillez cocher le chiffre (de 0 à 10) qui reflète le mieux votre réponse à la question suivante.**

**Cette question porte sur ce que vous avez ressenti pour la personne âgée que vous avez évaluée.**

Comment décririez-vous :

63- Son état de santé global ? **(Q63)**

0 1 2 3 4 5 6 7 8 9 10

(Aussi mauvais que possible - Aussi bon que possible)

64- Son état de santé par rapport aux personnes de son âge ? **(Q64)**

0 1 2 3 4 5 6 7 8 9 10

(Aussi mauvais que possible - Aussi bon que possible)

65- A la lumière de cet entretien, pensez vous qu’il y ait un risque de dégradation de son état de santé dans l’année à venir ? **(Q65)**

0 1 2 3 4 5 6 7 8 9 10

(Très grande chance Très peu de chance)
