# Supplementary material for: Development and validation of the FRAGIRE tool for assessment an older person’s risk for frailty
Source: BMC Geriatr. 2016 Nov 17;16:187. doi: 10.1186/s12877-016-0360-9 (PMC5114762; doi:10.1186/s12877-016-0360-9)
Supplement: Additional file 4: — The final FRAGIRE grid (A) English version and (B) Original version in French. (DOCX 45 kb) [file 12877_2016_360_MOESM4_ESM.docx]

The final FRAGIRE grid (**A)** English version and (**B)** Original version in French

1. English version

*Please mark the number (from 0 to 10) that best reflects your answer to the following questions.*

*Each question refers to how you have felt during the last week and how you feel today.*

*How would you describe:*

*How you describe your health status*? (Q1)

**Dimension GLOBAL HEALTH STATUS – Physiological well-being**

0 1 2 3 4 5 6 7 8 9 10

(0 -the worst health you can imagine,10 - the best health you can imagine)

How many times have you been hospitalized within the last 6 months? (Q4)

0 / 1-2 times / more than 2 / I don’t know

How you describe your general well-being? (Q5)

**Dimension Psychological – Depression, enjoyement, suicide**

0 1 2 3 4 5 6 7 8 9 10

(0 -the worst health you can imagine,10 - the best health you can imagine)

*Circle the best answer from those proposed*

In the last months:

Have you been happy with life in general? (Q8)

1 Not at all / 2 A little/ 3 Quite a bit/ 4 Very much

Have you been tired during the day**? (Q16)**

1 Not at all / 2 A little/ 3 Quite a bit/ 4 Very much

Have you suffered so much that you start having suicide ideation**? (Q19)**

0 No / 1 Yes

*The answer to this question will not be included in the score. In case of positive response, examiner may provide guidance or warning*

**Tests:**

**Dimension COGNITIVE**

The Isaac set test (STI),

The serious mental illnesses test (SMI).

*This dimension will not be included in the score but* *these tests will give the indications to consider in the evaluation of fragility.*

**Dimension ENVIRONMENTAL**

*Circle the best answer from those proposed:*

Have you felt lonely or abandonment**? (Q24)**

1 Not at all / 2 A little/ 3 Quite a bit/ 4 Very much

Your financial situation seemed sufficient to meet your needs**? (Q30)**

1 Not at all / 2 A little/ 3 Quite a bit/ 4 Very much

**Dimension SOCIO-CULTURAL**

*Circle the best answer from those proposed:*

*Do you use internet***? (Q31)**

1 Not at all / 2 A little/ 3 Quite a bit/ 4 Very much

Do you participate in sport activities (sport, art, etc.)**? (Q32)**

1 Not at all / 2 A little/ 3 Quite a bit/ 4 Very much

*Circle the best answer from those proposed:*

**Dimension SEXUAL**

Are you troubled by signs of aging**? (Q34)**

1 Not at all / 2 A little/ 3 Quite a bit/ 4 Very much

Are you interested in sexual activity**? (Q36)**

1 Not at all / 2 A little/ 3 Quite a bit/ 4 Very much

C*ircle the best answer from those proposed:*

**Dimension BURDEN OF HELP**

*Do you assist relative(s) you feel responsible for***? (Q37 & Q38)**

1 Not at all / 2 A little bit / 3 Quite a bit/ 4 Very much

**Dimension NUTRITIONAL – Taste, appetit, denture**

*Circle the best answer from those proposed*

In the last weeks:

Have you had problem with taste**? (Q40)**

1 Not at all / 2 A little bit / 3 Quite a bit/ 4 Very much

How many dental consultations have you had**? (Q44)**

0 / 1 / more than 1

*Circle the best answer from those proposed*

**Dimension MOBILITY**

Have you fallen in the last 6 months**? (Q54)**

0 / 1 / more than once

Have you had difficulties doing some physical activities such as carrying a loaded shopping bag or suitcase**? (Q55)**

1 Not at all / 2 A little bit / 3 Quite a bit/ 4 Very much

**Test:** walking speed 4m with 3 steps **(Q56)**

- normal ≥ 1 m/s
- impairment of balance and walking: between 0.65 and < 1 m/s

Deficiency (global): < 0.65 m/s

**Section for EXAMINER**

*Please mark the number (from 0 to 10) that best reflects your answer to the following questions.*

*Each question refers to how you felt about the older patient you have evaluated*

*How do you describe her/his global health status***? (Q63)**

0 1 2 3 4 5 6 7 8 9 10

(0- the worst health you can imagine,10 - the best health you can imagine)

**Points to be reviewed:**

Reformulation of the questions concerning information for the recognition of the taste of food and the health status to be more explicit;
- Reformulation of the general questions in the third person singular using the word "the applicant", for example: "Have the applicant participated in activities (sport, art ...)"?

1. Original version in French

Le comité d'experts suggère l'utilisation d'une réglette (type réglette d'évaluation de la douleur) pour la réponse aux questions de ce type (0 à10).

*Veuillez cocher le chiffre (de 0 à 10) qui reflète le mieux votre réponse à la question suivante.*

**Dimension ETAT DE SANTE GLOBAL – ressenti physiologique**

*Cette question porte sur ce que vous avez ressenti au cours de la semaine qui vient de s’écouler, aujourd’hui compris.*

**Comment décririez-vous votre état de santé ? (Q1)**

**0 1 2 3 4 5 6 7 8 9 10**

(0 Aussi mauvais que possible - 10 Aussi bon que possible)

*Entourez la réponse qui convient le mieux parmi les celles proposées*

**Combien de fois avez-vous été hospitalisé(e) au cours des 6 derniers mois ? (Q4)**

**0 / 1 à 2 fois / Plus de 2 fois / Ne sait pas**

**Dimension PSYCHIQUE – Dépression, plaisir, suicide**

*Veuillez cocher le chiffre (de 0 à 10) qui reflète le mieux votre réponse à la question suivante.*

*Cette question porte sur ce que vous avez ressenti au cours de la semaine qui vient de s’écouler, aujourd’hui compris.*

**Comment décririez-vous votre bien-être général ? (Q5)**

0 1 2 3 4 5 6 7 8 9 10

(0 Aussi mauvais que possible - 10 Aussi bon que possible)

*Entourez la réponse qui vous convient le mieux parmi celles proposées*

**Au cours du dernier mois :**

**Etes-vous heureux(se) la plupart du temps ? (Q8)**

1 Pas du tout / 2 Un peu / 3 Assez / 4 Beaucoup

**Vous sentez-vous fatigué(e) pendant la journée ? (Q16)**

1 Pas du tout / 2 Un peu / 3 Assez / 4 Beaucoup

**Souffrez-vous au point d’avoir des idées de suicide ? (Q19)**

0 Non / 1 Oui

*La réponse à cette question ne sera pas comprise dans le score. En cas de réponse positive, les évaluateurs pourront prévoir une orientation ou une alerte.*

**Dimension COGNITIVE**

**Tests :**

Set test d'Isaac (STI),

Score de Mémoire avec Indiçage (SMI).

*Cette dimension ne sera pas comprise dans le score mais les tests donneront des indications à prendre en compte dans l'évaluation de la fragilité.*

**Dimension ENVIRONNEMENTALE**

Entourez la réponse qui vous convient le mieux parmi celles proposées

**Avez-vous un sentiment de solitude et/ou d’abandon ? (Q24)**

1 Pas du tout / 2 Un peu / 3 Assez / 4 Beaucoup

**Votre niveau de ressources vous semble-t-il suffisant ? (Q30)**

1 Pas du tout / 2 Un peu / 3 Assez / 4 Beaucoup

**Dimension AIDANTS**

**Dimension SEXUELLE**

**Dimension SOCIO-CULTURELLE**

*Entourez la réponse qui vous convient le mieux parmi celles proposées*

**Utilisez-vous Internet ? (Q31)**

1 Pas du tout / 2 Un peu / 3 Assez / 4 Beaucoup

**Participez-vous à des activités (clubs sportif, artistique…) ? (Q32)**

1 Pas du tout / 2 Un peu / 3 Assez / 4 Beaucoup

*Entourez la réponse qui vous convient le mieux parmi celles proposées*

**Etes-vous affecté(e) par des signes visibles du vieillissement ? (Q34)**

1 Pas du tout / 2 Un peu / 3 Assez / 4 Beaucoup

**Vous intéressez-vous à la sexualité ? (Q36)**

1 Pas du tout / 2 Un peu / 3 Assez / 4 Beaucoup

**Dimension AIDANTS**

*Entourez la réponse qui vous convient le mieux parmi celles proposées*

**Vous occupez-vous d’un proche dont vous vous sentez responsable ?  (Q37 & Q38)**

1 Pas du tout / 2 Un peu / 3 Assez / 4 Beaucoup

**Dimension MOTRICITE**

**Dimension NUTRITIONNELLE – Goût, appétit, bucco-dentaire**

*Entourez la réponse qui vous convient le mieux parmi celles proposées*

**Ces dernières semaines, avez-vous des difficultés pour reconnaître le goût des aliments que vous consommez ? (Q40)**

1 Pas du tout / 2 Un peu / 3 Assez / 4 Beaucoup

**De combien de consultations dentaires annuelles avez-vous bénéficié? (Q44)**

0 / 1 / plus de 1

*Entourez la réponse qui vous convient le mieux parmi celles proposées*

**Avez-vous chuté au cours des 6 derniers mois ? (Q54)**

0 / 1 / plus de 1

**Ces dernières semaines, avez-vous des difficultés à faire certains efforts physiques pénibles comme porter un sac à provisions chargé ou une valise ? (Q55)**

1 Pas du tout / 2 Un peu / 3 Assez / 4 Beaucoup

**Test :** vitesse de marche sur 4 m avec 3 seuils :  **(Q56)**

normale ≥ 1 m/s

altération de l'équilibre et de la marche : entre 0,65 et < 1 m/s

fragilité (globale) : < 0,65 m/s

**PARTIE EVALUATEUR**

*Veuillez cocher le chiffre (de 0 à 10) qui reflète le mieux votre réponse à la question suivante. Cette question porte sur ce que vous avez ressenti pour la personne âgée que vous avez évaluée.*

**Comment décririez-vous son état de santé global ?**   **(Q63)**

0 1 2 3 4 5 6 7 8 9 10

(Aussi mauvais que possible - Aussi bon que possible)

**Eléments à revoir :**

- Reformulation des questions afférentes à la reconnaissance du goût des aliments et à l'état de santé afin d'être plus explicites ;
- Reformulation générale des questions à la troisième personne du singulier avec comme sujet "le demandeur", par exemple : "Le demandeur participe-t-il à des activités (club sportif, artistique…) ? »
