# Supplementary material for: Development and validation of the FRAGIRE tool for assessment an older person’s risk for frailty
Source: BMC Geriatr. 2016 Nov 17;16:187. doi: 10.1186/s12877-016-0360-9 (PMC5114762; doi:10.1186/s12877-016-0360-9)
Supplement: Additional file 5: — Details on the construction of the prognostic score. (DOCX 46 kb) [file 12877_2016_360_MOESM5_ESM.docx]

**Details on the construction of the prognostic score**

$$\begin{matrix} \mathbf{Raw Prognostic score}_{logistic model}= \begin{matrix} \begin{matrix} \begin{matrix} \\ 6.4420+ \\ \boldsymbol{Q}\boldsymbol{1} \times\left( -0.0484 \right)+ \boldsymbol{Q}\boldsymbol{4} \times\left( +0.0832 \right)+ \\ \boldsymbol{Q}\boldsymbol{5} \times\left( -0.2624 \right)+ \boldsymbol{Q}\boldsymbol{8} \times\left( -0.0839 \right)+ \boldsymbol{Q}\boldsymbol{16} \times(0.0114) + \\ \boldsymbol{Q}\boldsymbol{24} \times\left( 0.5412 \right)+ \boldsymbol{Q}\boldsymbol{30} \times(-0.5680) + \\ \boldsymbol{Q}\boldsymbol{31} \times\left( -0.8464 \right)+\boldsymbol{Q}\boldsymbol{32} \times(-0.4330) + \end{matrix} \\ \boldsymbol{Q}\boldsymbol{37-38}*(-0.0762) + \\ \boldsymbol{Q}\boldsymbol{40} \times\left( +0.2703 \right)+ \boldsymbol{Q}\boldsymbol{44} \times(-0.4621) + \end{matrix} \\ \boldsymbol{Q}\boldsymbol{54} \times\left( 0.2741 \right)+ \boldsymbol{Q}\boldsymbol{56} \times\left( 0.1038 \right)+\boldsymbol{Q}\boldsymbol{55} \times\left( 0.0367 \right)+ \end{matrix} \\ \boldsymbol{Q}\boldsymbol{63} \times(-0.1550) \end{matrix}$$

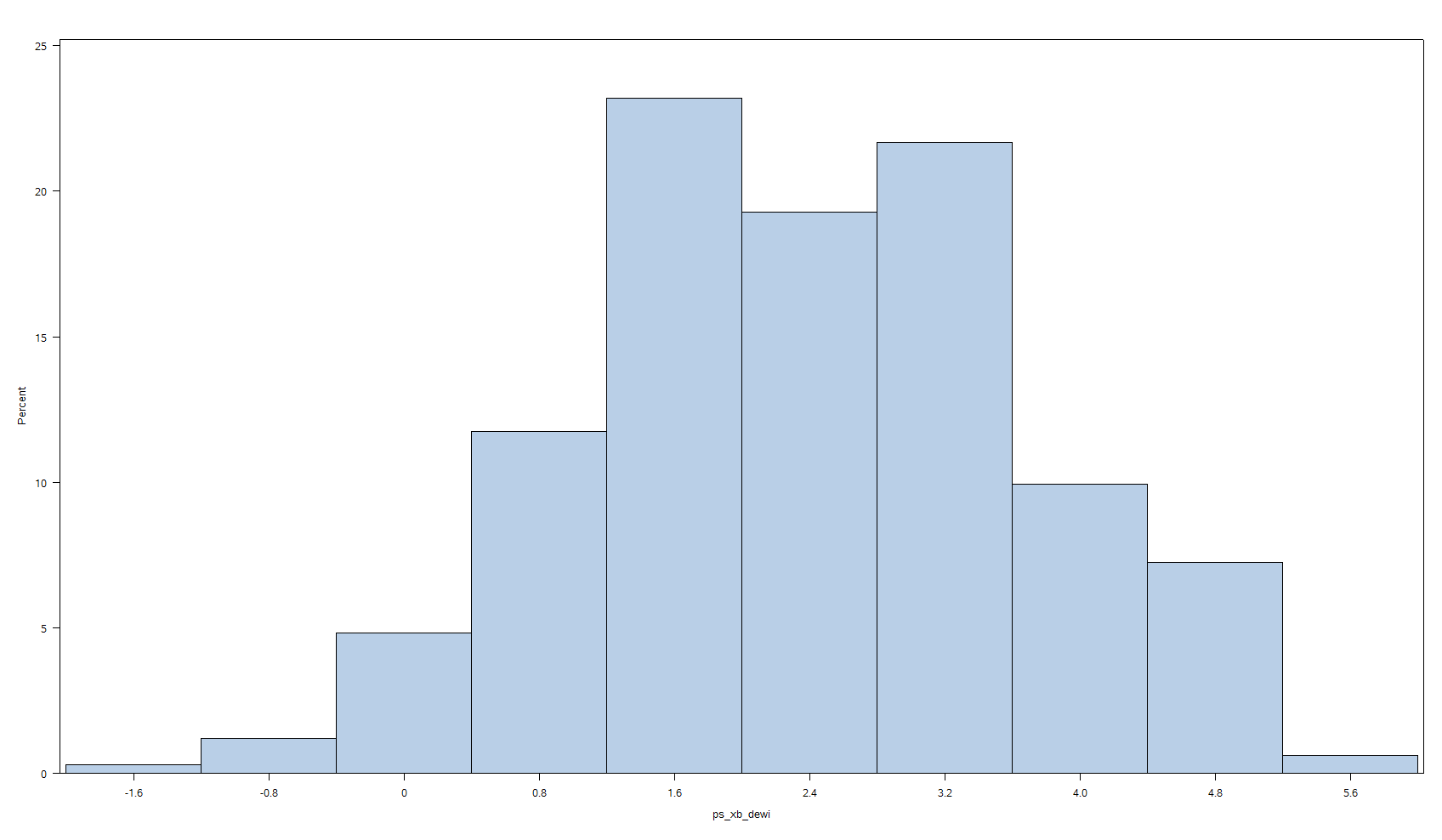
$\mathbf{Raw Prognostic score}_{logistic model} \in\left[ -6.1236; 8.4415 \right]$ $:theorical range$

***Descriptive statistics:***

| N | 339 |
| --- | --- |
| N missing | 48 |
| Min | -3.20 |
| Max | 6.28 |
| Median | 2.05 |
| Mean | 1.99 |
| Std | 1.54 |

$$\mathbf{Normalized Prognostic score}_{\mathbf{logistic model}}= \frac{\left( 100-0 \right)\times\left( {Raw Prognostic score}_{logistic model}--6.1236 \right)}{8.4415--6.1236}+0$$

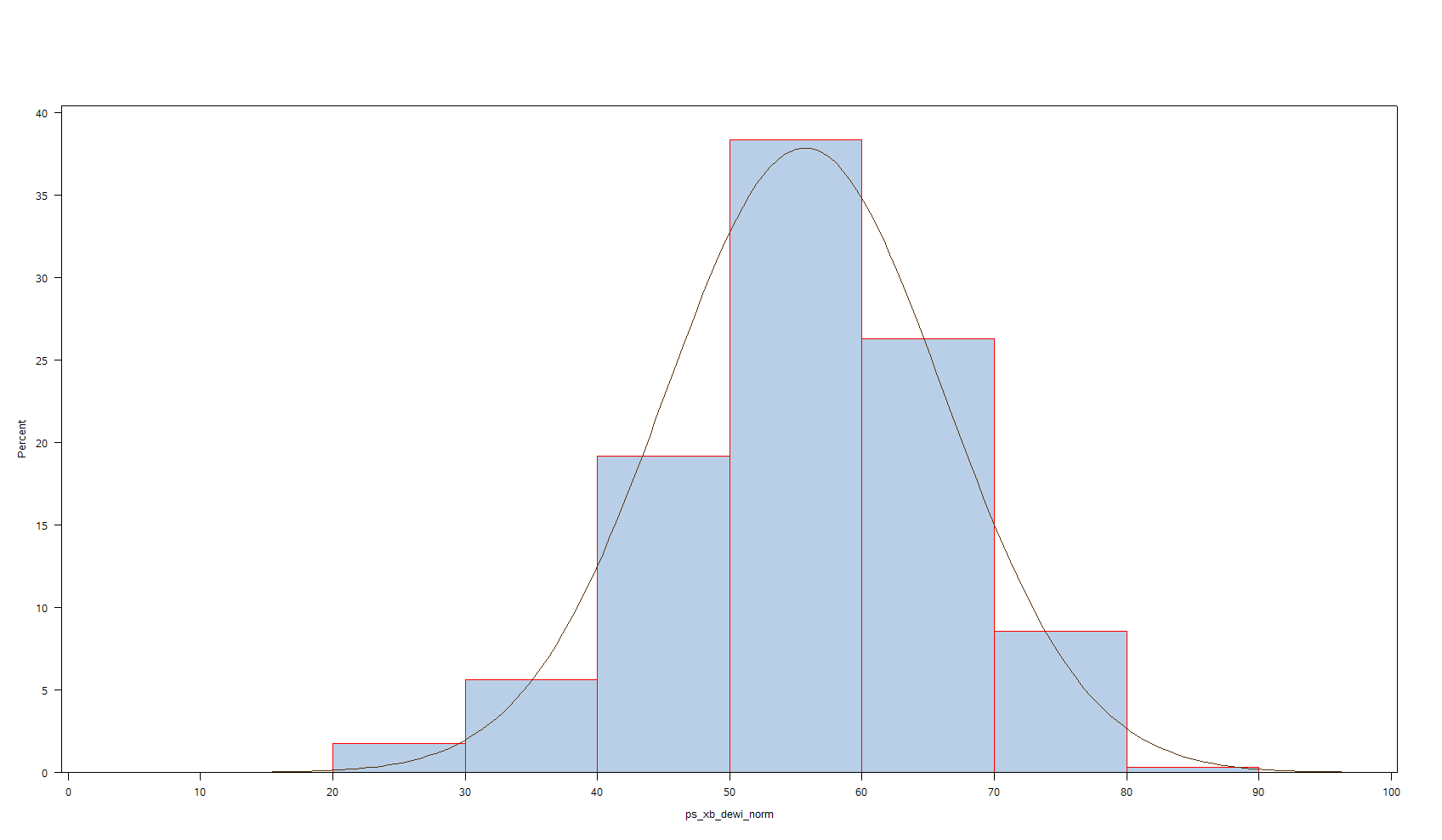
$\mathbf{Normalized Prognostic score}_{\mathbf{logistic model}} \in\left[ 0-100 \right]$

***Descriptive statistics:***

| N | 339 |
| --- | --- |
| Mean | 55.7 |
| Std | 10.5 |
| Min | 20.1 |
| Max | 85.1 |
| Median | 56.1 |
| IQR range | 49.6-62.6 |
