# Supplementary material for: Development and validation of the FRAGIRE tool for assessment an older person’s risk for frailty
Source: BMC Geriatr. 2016 Nov 17;16:187. doi: 10.1186/s12877-016-0360-9 (PMC5114762; doi:10.1186/s12877-016-0360-9)
Supplement: Additional file 6:Table S1. — Description of the Memory Impairment Screen results in the overall population and according to the financially helped status at day 0 and day 3. (DOCX 15 kb) [file 12877_2016_360_MOESM6_ESM.docx]

**Table S1:** Description of the Memory Impairment Screen results in the overall population and according to the financially helped status at day 0 and day 3

|  | | | **Population Total**  **N (%)** | | **Non-financially helped group**  **N (%)** | | **Financially helped group**  **N (%)** | | ***P* value** |
| --- | --- | --- | --- | --- | --- | --- | --- | --- | --- |
| **MIS day 0** | | |  | |  | |  | |  |
| Not performed | | | 13 (3.4) | | 0 (0.0) | | 13 (3.8) | | **.06** |
| Performed | | | 372 (96.6) | | 47 (100) | | 325 (96.1) | |  |
| **MIS day 3** | | |  | |  | |  | |  |
| Not performed | | | 48 (12.5) | | 4 (8.5) | | 44 (13.0) | | .35 |
| Performed | | | 337 (87.5) | | 43 (91.5) | | 294 (87.0) | |  |
| **MIS** | | |  | |  | |  | |  |
| Not performed | | | 8 (2.1) | | 0 (0.0) | | 8 (2.4) | | .29 |
| Day 0 | | | 40 (10.4) | | 4 (8.5) | | 36 (10.6) | |  |
| Day 3 | | | 5 (1.3) | | 0 (0.0) | | 5 (1.5) | |  |
| Day 0 + day 3 | | | 332 (86.2) | | 43 (91.5) | | 289 (85.5) | |  |
| **Score MIS day 0** | | |  | |  | |  | |  |
| N | | | 372 | | 47 | | 325 | | .99 |
| Mean | | | 6.49 | | 6.48 | | 6.49 | |  |
| Standard deviation | | | 1.82 | | 1.74 | | 1.84 | |  |
| Median (min-max) | | | 7 (1-12) | | 7 (1-8) | | 7 (1-12) | |  |
| **Score MIS day 3** | | |  | |  | |  | |  |
| N | | | 337 | | 43 | | 294 | | .72 |
| Mean | | | 7.16 | | 7.09 | | 7.17 | |  |
| Standard deviation | | | 1.39 | | 1.39 | | 1.39 | |  |
| Median (min-max) | | | 8 (2-12) | | 8 (2-8) | | 8 (2-12) | |  |
|  | |  |  | |  | |  | |  |

MIS : Memory Impairment Screen
