# Supplementary material for: Development and validation of the FRAGIRE tool for assessment an older person’s risk for frailty
Source: BMC Geriatr. 2016 Nov 17;16:187. doi: 10.1186/s12877-016-0360-9 (PMC5114762; doi:10.1186/s12877-016-0360-9)
Supplement: Additional file 7: Table S2. — Description of the Isaacs Set Test results in the overall population and according to the financially helped status. (DOCX 15 kb) [file 12877_2016_360_MOESM7_ESM.docx]

**Table S2:** Description of the Isaacs Set Test results in the overall population and according to the financially helped status.

|  | **Population Total**  **N (%)** | **Non-financially helped group**  **N (%)** | **Financially helped group**  **N (%)** | ***P value*** |
| --- | --- | --- | --- | --- |
| **IST day 0** |  |  |  |  |
| Performed | 6 (1.6) | 1 (2.1) | 5 (1.5) | .74 |
| Not performed | 379 (98.4) | 46 (97.9) | 333 (98.5) |  |
| **IST day 3** |  |  |  |  |
| Not performed | 45 (11.7) | 4 (8.5) | 41 (12.1) | .45 |
| Performed | 340 (88.3) | 43 (91.5) | 297 (87.9) |  |
| **IST** |  |  |  |  |
| Not performed | 4 (1.0) | 1 (2.1) | 3 (0.9) | .54 |
| Day 0 | 41 (10.7) | 3 (6.4) | 38 (11.2) |  |
| Day 3 | 2 (0.5) | 0 | 2 (0.6) |  |
| Day 0 + day 3 | 338 (87.8) | 43 (91.5) | 295 (87.3) |  |
| **Score IST day 0** |  |  |  |  |
| N | 379 | 46 | 333 | **.001** |
| Mean | 30 | 33.3 | 29.6 |  |
| Standard deviation | 7.37 | 7.58 | 7.23 |  |
| Median (min-max) | 29 (13-58) | 33.5 (20-52) | 29 (13-58) |  |
| **Score IST day 3** |  |  |  |  |
| N | 340 | 43 | 297 | **.001** |
| Mean | 30.5 | 33.9 | 29.9 |  |
| Standard deviation | 7.34 | 8.62 | 7.01 |  |
| Median (min-max) | 30 (14-56) | 35 (17-56) | 29 (14-54) |  |

IST: Isaacs Set Test
